# A randomized placebo-controlled trial of an omega-3 fatty acid and vitamins E+C in schizophrenia

**DOI:** 10.1038/tp.2013.110

**Published:** 2013-12-17

**Authors:** H Bentsen, K Osnes, H Refsum, D K Solberg, T Bøhmer

**Affiliations:** 1 Center for Psychopharmacology, Diakonhjemmet Hospital, Oslo, Norway; 2 Division of Psychiatry, Oslo University Hospital, Aker, Oslo, Norway; 3 Department of Psychosomatic Medicine, Oslo University Hospital, Rikshospitalet, Oslo, Norway; 4 Nutritional Laboratory, Department of Medical Biochemistry, Oslo University Hospital, Aker, Oslo, Norway

**Keywords:** alpha-tocopherol, ascorbic acid, clinical trial, delusions, eicosapentaenoic acid

## Abstract

Membrane lipid metabolism and redox regulation may be disturbed in schizophrenia. We examined the clinical effect of adding an omega-3 fatty acid and/or vitamins E+C to antipsychotics. It was hypothesized that lower baseline levels of polyunsaturated fatty acids (PUFAs) would predict more benefit from the add-on treatment. The trial had a multicenter, randomized, double-blind, placebo-controlled 2 × 2 factorial design. Patients aged 18–39 years with schizophrenia or related psychoses were consecutively included at admission to psychiatric departments in Norway. They received active or placebo ethyl-eicosapentaenoate (EPA) 2 g day^−1^ and active or placebo vitamin E 364 mg day^−1^+vitamin C 1000 mg day^−1^ (vitamins) for 16 weeks. The main outcome measures were Positive and Negative Syndrome Scale (PANSS) total and subscales scores, analyzed by linear mixed models. Ninety-nine patients were included. At baseline, erythrocyte PUFA were measured in 97 subjects. Given separately, EPA and vitamins increased drop-out rates, whereas when combined they did not differ from placebo. In low PUFA patients, EPA alone impaired the course of total PANSS (Cohen's *d*=0.29; *P*=0.03) and psychotic symptoms (*d*=0.40; *P*=0.003), especially persecutory delusions (*d*=0.48; *P*=0.0004). Vitamins alone impaired the course of psychotic symptoms (*d*= 0.37; *P*=0.005), especially persecutory delusions (*d*=0.47; *P*=0.0005). Adding vitamins to EPA neutralized the detrimental effect on psychosis (interaction *d*=0.31; *P*=0.02). In high PUFA patients, there were no significant effects of trial drugs on PANSS scales. In conclusion, given separately during an acute episode, EPA and vitamins E+C induce psychotic symptoms in patients with low levels of PUFA. Combined, these agents seem safe.

## Introduction

The mainstay of treatment in schizophrenia, by dopamin D_2_ receptor antagonists, is insufficient and bedevilled by adverse effects.^1^ Authors reviewing ‘emerging drugs' concluded: ‘…add-on strategies to already available antipsychotics are the most promising option'. Moreover, ‘drugs targeting differential subsyndromes of the disorder will probably offer more auspicious opportunities' than aiming ‘one magic bullet for the treatment of schizophrenia'.^2^ Abnormalities in membrane lipids and redox regulatory systems may account for a multitude of schizophrenia's manifestations, and may be targets for preventive and therapeutic interventions.^3, 4, 5, 6^ *Either* polyunsaturated fatty acids (PUFA)^7,8^ *or* redox regulators, such as vitamins E or C,^9,10^ ginkgo biloba^11^ or n-acetyl-cysteine,^12^ have been used in randomized clinical trials. Assuming that omega-3 fatty acids and antioxidants would act synergistically, we undertook a randomized placebo-controlled trial with ethyl-eicosapentaenoate (ethyl-EPA) and vitamins E+C. We have reported that levels of PUFA at baseline are bimodally distributed,^6^ defining two clinically distinct types of schizophrenia (low and high PUFA groups).^13^ In the present part of the study, we hypothesized that improvement of schizophrenia symptoms would be stronger if EPA or vitamins E+C were added to antipsychotic drugs, that the combination of trial drugs would be even better, and that lower levels of erythrocyte (RBC) PUFA at baseline would predict more benefit from trial drugs.

## Materials and methods

### Study design and participants

This was a phase IIB-III add-on clinical trial with a multicenter, randomized, double-blind, placebo-controlled, fixed dose, 2 × 2 factorial design. The trial was conducted according to the ICH Harmonised Tripartite Guideline for Good Clinical Practice.^14^ The full trial protocol can be accessed at www.psykofarmakologi.no. The project protocol was approved by the Regional Committee of Medical Research Ethics, Health Region East, Norway (ref. 302–02–00060). Informed consent was obtained from all patients included in the study. Patients with schizophrenia, schizoaffective disorder or schizophreniform disorder (DSM-IV), aged 18–39 years, admitted to a psychiatric department at nine Norwegian hospitals within the previous 21 days, prescribed antipsychotics, with no substance dependence (DSM-IV), no known allergy to trial agents, no warfarin currently and no anamnestic indicators of impaired hemostasis were included. All investigators were physicians. The clinical trial is registered at http://clinicaltrials.gov/ct2/show/NCT00419146.

### Treatment

Patients were allocated to four treatment groups by simple randomization (computerized random number generator). A block randomization, entailing equally large treatment groups, was requested, but not executed because of a misunderstanding. A pharmacist enclosed assignments in sequentially numbered opaque envelopes, kept at her pharmacy. Antipsychotic drugs were prescribed at therapists' choice. Compliance with this medication was assessed at each visit by asking the patient or, if in doubt, the therapist. In addition, patients received from the first visit twice a day two capsules of active or placebo ‘EPA' and twice a day two tablets of active or placebo α-tocopherol-ascorbic acid (‘vitamins') for 16 weeks. Each ‘EPA' capsule contained either 500 mg ethyl eicosapentaenoate (Ethyl-EPA) with 1 mg α-tocopherol (‘active') or 500 mg paraffin oil (‘placebo') (Laxdale Ltd., Stirling, UK). Each ‘Vitamins' tablet contained either 91 mg (136 IU) RRR-α-tocopherol and 250 mg slow release ascorbic acid (CellaVie) (‘active') or a similar amount of dicalciumphosphate (‘placebo') (Ferrosan A/S, Søborg, Denmark). Thus, participants belonged to Group 1 (double placebo), Group 2 (active vitamins), Group 3 (active EPA) or Group 4 (double active). Trial drugs were supplied in opaque plastic bottles kept at room temperature. Ethyl-EPA capsules remain stable in these bottles for 12 months at 25 °C/60% relative humidity. Every month drugs was transferred to a dosage box. A patient's compliance was assessed by counting the number of capsules and tablets remaining in the box. Patients, healthcare providers, and investigators did not know treatment assignment. A questionnaire filled in after the last session indicated no links between guess and assignment among patients (*n*=20) and investigators (*n*=4).

### Outcome

Patients were assessed at five visits with 4 weeks intervals. The baseline visit took place within 4 weeks after hospital admission. Assessment methods are described at http://www.nature.com/tp (Supplementary Text).^6,13^ The main clinical instrument was the Positive and Negative Syndrome Scale (PANSS) – Structured Interview Version, applied at the start, after 8 and 16 weeks. Adverse events were assessed at each visit with the UKU Side Effect Rating Scale (USERS). Vital signs and biochemical variables were measured at the first and the last visit. The primary outcome measure was the change in total PANSS. The secondary outcome measures were changes in each of the PANSS subscales. All other measures were considered exploratory.

### Statistical analyses

The planned sample size *n*=200 was based on a moderate standardized effect size (=0.5) of ethyl-EPA on total PANSS, an *α* error level=0.05 (two-tailed), a statistical power=0.88 and 25% drop-outs. The obtained sample size was 99 (power=0.70). Analyses were done with the PASW Statistics 18 program (SPSS Inc, Chicago, IL, USA/IBM, New York, USA). The *α*-level was set at 0.05 (two-tailed). All longitudinal analyses were performed primarily on the intent-to-treat (ITT) sample, secondarily on the per protocol (PP) sample, that is, the patients with both complete follow-up (that is, no drop-out) and study drug compliance. According to the protocol, we used linear mixed models.^15,16^ Missing values were assumed to be ignorable, that is, ‘missing at random'. This is a likely assumption for randomized trials.^16^ Missing at random is compatible with linear mixed model analyses.^17^

The following random intercept model equation (Model 2) was used in order to test our hypotheses, taking into account the influence of baseline PUFA (as a continuous variable) on the effects of treatment: *Y_ij_*=*β*_0_+*β*_1_ Time*_ij_*+*β*_2_ (Time*_ij_* × Vitamins*_i_*)+*β*_3_ (Time*_ij_* × EPA*_i_*)+*β*_4_ (Time*_ij_* × Vitamins*_i_* × EPA*_i_*)+*β*_5_ PUFA*_i_*+*β*_6_ (PUFA*_i_* × Time*_ij_* × Vitamins*_i_*)+*β*_7_ (PUFA*_i_* × Time*_ij_* × EPA*_i_*)+*β*_8_ (PUFA*_i_* × Time*_ij_* × Vitamins*_i_* × EPA*_i_*)+*b_i_*+*e_ij_*. Here *Y_ij_* is the response matrix of the *i*^th^subject, *β*_0_ the population mean *y*-intercept (that is, the estimated effect at baseline, adjusted for PUFA), *β*_2_-*β*_8_ the fixed effects, *b_i_* the *i*^th^subject's deviation from *β*_0_ (that is, random intercept) and *e_ij_* the within-subject measurement error. Time*_ij_* was coded as 0 at baseline, and (1), (1,2) or (1,2,3,4) at follow-up, depending on the number of repeated assessments. In this paper, all *β*s are transformed to representing a period of 16 weeks. PUFA is RBC PUFA (μg g^−1^) at baseline. Vitamins*_i_* and EPA*_i_* were coded as 0=placebo, 1=active. The vectors of fixed effects (*β*) were estimated by restricted maximum likelihood analysis. Statistical inferences about *β* were done by a Wald test (*α* level=0.05). We used unstructured component covariance matrices.^16^ Standardized effect sizes (Cohen's *d*) were calculated as *d*=2 *√(F/df)*, where *F* is the F test statistic for the effect of interest and *df* the corresponding degrees of freedom. If none of the interaction effects involving PUFA were statistically significant, a simplified model (Model 1) was used to estimate time × treatment effects: *Y_ij_*=*β*_0_+*β*_1_ Time*_ij_*+*β*_2_ (Time*_ij_* × Vitamins*_i_*)+*β*_3_ (Time*_ij_* × EPA*_i_*)+*β*_4_ (Time*_ij_* × Vitamins*_i_* × EPA*_i_*)+*b_i_*+*e_ij_*. For the purpose of illustration, we estimated for all variables the time × treatment effects at different levels of baseline PUFA. Previously, we have shown a bimodal distribution of baseline PUFA, defining two normally distributed subgroups.^6^ We centered the PUFA level at the mean of the low PUFA group (102 μg g^−^^1^ RBC) and the mean of the high PUFA group (442 μg g^−1^ RBC), thus replacing PUFA in Model 2 by (PUFA – mean of PUFA group). Assuming the latter variable to be zero, we showed effects of treatment in a typical low (Model 3A) and a typical high (Model 3B) PUFA patient, respectively.

Adjusting for multiple testing by Bonferroni correction was only done for the confirmatory analysis of time × treatment effects on the three PANSS subscales (that is, adjusted threshold *P*=0.016).^18,19^ For the rest, adjustment was *not* considered appropriate. Except for the confirmatory analysis of the primary outcome measure (total PANSS), these analyses should be considered as exploratory.

We performed several sensitivity analyses. All variables were tested in the PP group of patients. For the variables with the strongest effects only, we added the following sensitivity analyses. In order to ensure the generalizability of our linear mixed model findings, we replaced the unstructured covariance matrix by the more conservative compound symmetry. Because the trial groups were small and thus prone to sample selection bias, we analyzed the main significant findings by a resampling method, that is, non-parametric bootstrapping with bias-corrected and accelerated (BC_a_) confidence intervals.^20,21^ We repeated sampling with replacement from the original sample 9999 times, creating bootstrap samples better representing the population from which our original sample was drawn. In order to check the missing at random assumption, thus that the probability of an observation being missing should depend only on observed data, we assessed predictors of drop-out by logistic regression.^17,22^ We also performed ‘worst case analysis', assigning the best possible outcome (that is, the most extreme value in the study) to missing values in the placebo group and the worst possible outcome to those of the experimental groups.^17^

## Results

### Participants

**Figure 1:**
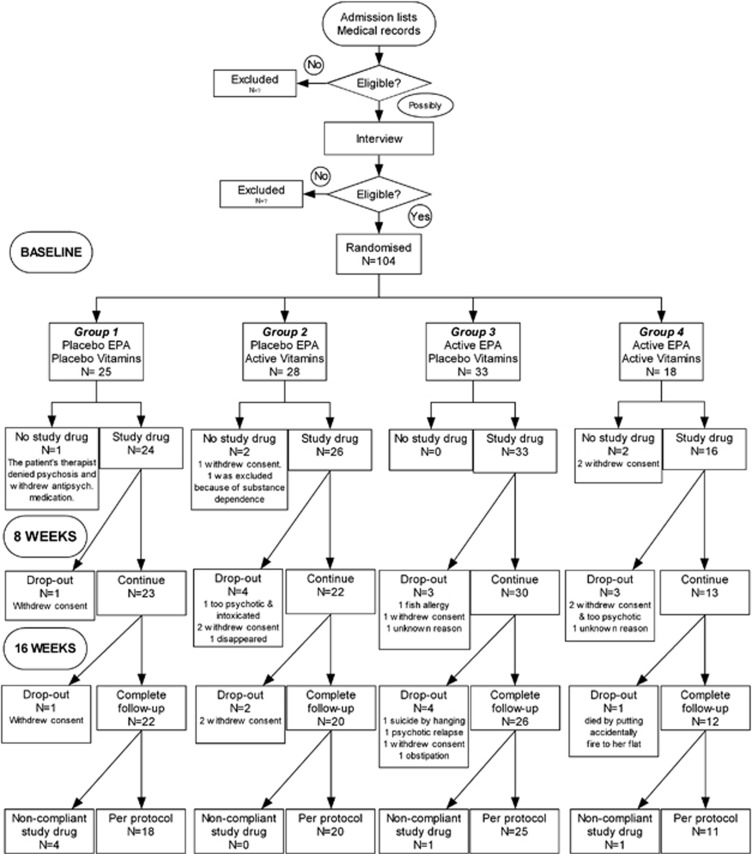
Enrollment, randomization and follow-up of study participants. EPA, ethyl-eicosapentaenoic acid; Vitamins, vitamin E+vitamin C. For practical reasons, it was impossible to assess the number of patients assessed for eligibility (thus, excluded *n*=?).

Figure 1 shows the flow of patients in the study. The actual sample size was less than the planned because rates of admission to hospital were overestimated, drug dependence prevalence was underestimated, the largest center had to be excluded because of no recruitment, and the enrollment had to be stopped by December 2003 for pragmatic reasons. Eligible participants were recruited from 14 September 2001–16 December 2003. One hundered and four patients were randomly assigned to the four treatment groups. Five patients did not fulfill inclusion criteria and were excluded before receiving any trial drugs. The remaining 99 patients constituted the ITT sample. 97 patients had RBC PUFA measured at baseline (ITT-PUFA), among whom 72 remained for PP analysis. Key characteristics for the four treatment groups are shown in Table 1 (for more variables, see Supplementary Table S1). Baseline data for the ITT sample and PUFA subgroups are reported at http://www.nature.com/tp (Supplementary Table S2).^6^ The mean age of the ITT sample was 27.4 years, 64% were male, 72% had schizophrenia and 20% schizoaffective disorder. The median duration of illness was 4 years, and 31% of the patients were hospitalized for the first time. After admission to hospital, all patients were prescribed antipsychotics.

Analyzing drop-out and PP status, we used PUFA as a binary variable, defining two groups of patients.^6^ In bivariate analysis, drop-out and PP status are best predicted by being a low PUFA patient and by complaining about adverse events at the 4 weeks session. Eleven out of 30 patients (37%) dropped out in the low PUFA group versus 8 out of 67 patients (12%) in the high PUFA group (*χ*^2^-test, *P*=0.007). 35 out of 51 (69%) patients complaining of adverse events after 4 weeks versus 38/42 (91%) of non-complainers completed the trial (PP) (Fisher's exact test, *P*=0.01). In a multiple logistic regression model, containing the predictors ‘complaining of adverse events after 4 weeks' (+), PUFA group (0=low, 1=high) (−), duration of illness (+), study center L (+), EPA (+), Vitamins (+) and EPA × Vitamins (−), all variables are significantly predicting drop-out (+,−: sign of coefficient B; Hosmer and Lemeshow goodness-of-fit test: *χ*^2^=10.1, df= 8, *P*=0.26). EPA and vitamins, given separately, increase the risk of drop-out with odds ratios 43.3 (2.3–832; *P*=0.01) and 19.7 (1.1–336; *P*=0.04), respectively. By giving EPA and Vitamins together, the risk of drop-out does not differ from that of double placebo. In this model, drop-out is more likely in the low than in the high PUFA group (OR=10.8, 1.7–67.4, *P*=0.01). Including baseline PUFA as an effect modifier of the drug group variables entails overfitting of the model. Simplified models, with EPA × Vitamins excluded and with PUFA as a continuous variable, suggest that the lower the PUFA, the more EPA and Vitamins increase the risk of drop-out. Baseline severity of illness (PANSS total and subscales) does not predict loss to follow-up. Thus, the effect of baseline PUFA is not mediated by measured illness variables.

### Effects of trial drugs on mental status

**Figure 2:**
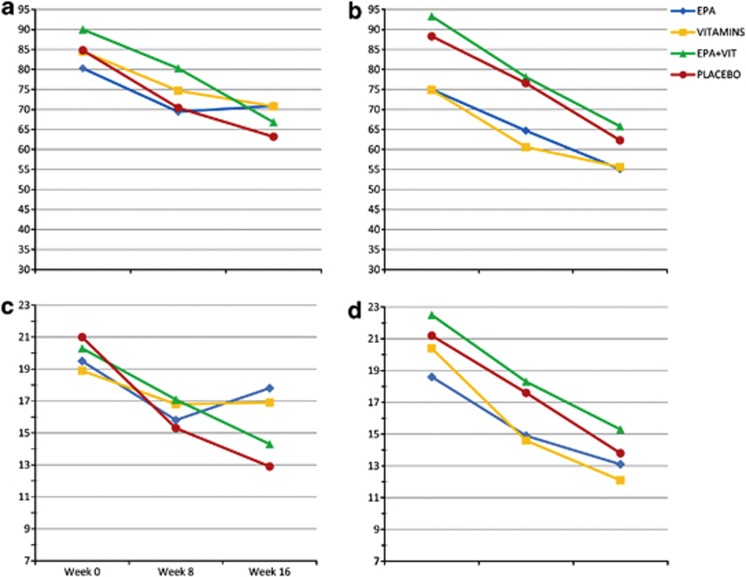
Course of schizophrenia symptoms by trial drugs and red blood cell fatty acids. Descriptive mean trajectories of PANSS scores over time in the four treatment groups (EPA=ethyl-eicosapentaenoic acid 2 g day^−1^, VITAMINS= vitamin E 364 mg day^−1^+vitamin C 1 g day^−1^, EPA+VIT= EPA and VITAMINS combined, PLACEBO= placebo EPA and placebo VITAMINS). **a** and **b** show the course of total PANSS scores in the low PUFAs (*n*=30) and high PUFA groups (*n*=67), respectively. **c** and **d** show the course of Positive subscale scores in the low PUFA and high PUFA groups, respectively. All patients were prescribed antipsychotic drugs. Missing values of PANSS (10.0%) were imputed by values predicted by linear mixed model analysis (Model 2).

Unless stated otherwise, the analyses involved the ITT-PUFA sample (*n*=97). According to Model 1 (see Statistical analysis), trial drugs do not change Total PANSS course (*P*>0.50). Following the protocol, we expanded Model 1 by adjusting for RBC PUFA at baseline, improving goodness-of-fit immensely (*χ*^2^=55.7; df=4; *P*=2 × 10^−11^) (Model 2, see Statistical analysis). Thus, it is essential to take baseline PUFA into account in order to have an optimal statistical model. In Model 2, EPA impairs the course of total PANSS (*P*=0.02). Bootstrapping shows this effect as a trend (*P*=0.08). The effects of all treatment terms are modified by PUFA at *P*<0.15 (Table 2). Centering the model at the average PUFA value of the low PUFA group, EPA alone increases severity of illness by 12.6 points (95% CI, 1.0–24.4; *P*=0.03) compared with placebo EPA (Model 3A, see Statistical analysis). Thus, imputing the estimated values into Model 2, on average total PANSS decreases from 80.8 to (80.8–22.4 × 1+9.2 × 0+12.6 × 1–22.8 × 0)=71.0 over 16 weeks with EPA (Group 3), compared with 58.4 when double placebo is given (Group 1) (Cohen's *d=*0.29). In the typical high PUFA patient, no effects are significant. Symptom courses are shown graphically in Figures 2a and b. Most treatment effects (Model 2) are stronger in PP analyses than in ITT analyses. In a ‘worst case analysis' (Model 2) both EPA and Vitamins impair outcome (*P*=0.01 and *P*<0.001, respectively). The effect of Vitamins, however, depends on PUFA (*P*=0.003). Using a compound symmetry gives practically the same results as an unstructured covariance matrix?

Among the PANSS subscales, trial drugs change the course of positive symptoms only. Regardless of PUFA (Model 1), EPA impairs this outcome non-significantly (*P*=0.09). In Model 2, where baseline PUFA is taken into account, EPA and Vitamins impair the course of positive symptoms (*P*=0.003 and *P*=0.002, respectively). Combining EPA and Vitamins neutralize this effect (*P*=0.01). Applying bootstrapping, all these effects are significant (*P*=0.04, *P*=0.02 and *P*=0.02, respectively). In Model 2, all time × treatment effects are modified by PUFA (*P*≤0.01) (Table 2). In a low PUFA patient (Model 3A), EPA or Vitamins impair highly significantly the course of positive symptoms (Table 2). Combining them neutralizes this detrimental effect. Thus, the effects of EPA-Vitamins and double placebo do not differ (*P*=0.79). On average, the Positive subscale score of a low PUFA patient in Group 1 decreases from 18.7 to 11.0, in Group 2 to 16.4, in Group 3 to 16.6, and in Group 4 to 12.0 (Table 2, Figure 2c). Cohen's *d*s are 0.40 (EPA), 0.37 (Vitamins) and 0.31 (the interactional effect). In the high PUFA patient, no effects are significant (Figure 2d). Most treatment effects (Model 2) are stronger in PP analyses than in ITT analyses. In ‘worst case analysis' (Model 2) both EPA and Vitamins impair outcome (*P*=0.001 and *P*<0.001, respectively), although the effect of Vitamins depends on PUFA (*P*<0.001). Using a compound symmetry gives practically the same results as an unstructured covariance matrix.

Among positive symptoms, Suspiciousness/persecution (P6) and Delusions (P1) are the most affected by trial drugs. In Model 2 for P6, all effects of treatment terms are highly significant, and all are significant in the corresponding bootstrap model. PUFA is a highly significant effect modifier (see Supplementary Table S4). In a typical low PUFA patient, *d* is 0.48 (*P*=0.0004) for EPA, *d* is 0.47 (*P*=0.0005) for Vitamins, and *d* is 0.03 (*P*=0.79) for EPA-Vitamins. Applying Model 2 for P1, delusions are increased by EPA and Vitamins, combining EPA and Vitamins neutralizes these effects, and PUFA modifies them (confirmed by bootstrapping, except for Vitamins) (see Supplementary Table S4). Beyond P6 and P1, EPA increases significantly hallucinatory behavior (P3), excitement (P4) and unusual thought content (G9), and Vitamins increase hostility (P7), G9 and preoccupation (G15).

### Other effects of trial drugs

Nine out of 99 patients experienced serious adverse events in the ITT group, and 6 of 74 in the PP group (Figure 1). Two patients died (groups 3 and 4). There is no link between treatment allocation and the number of serious adverse events (Fisher's exact tests, *P*>0.48). Trial drugs are not linked to total USERS adverse events (Table 2).

Vitamins increase the dosage of antipsychotics during the trial (Model 2, *P*=0.047). PUFA is a negative effect modifier (*P*=0.01) (Table 2). Thus, there is a trend that the low PUFA patient will use more antipsychotic drugs on active than on placebo Vitamins (*P*=0.096), whereas there will be no effect on dosage for the high PUFA patient (Table 2, Models 3A-B). EPA has no effect on dosage of antipsychotics (Model 2, *P*=0.85). Adjusted for treatment parameters, dosage is 0.4 DDD higher in the high than in low PUFA group (95% CI, 0.04–0.7; *P*=0.03). Seventy-one out of 74 (96%) PP patients used the same type of antipsychotics during the trial.^13^ Among the completers, 27% did not change dosage, 49% changed once or twice, and 22% changed three to five times. There were no differences between study groups in the number of changes.

Vitamins increase lipid-adjusted s-alpha-tocopherol (+6.2 μmol l^−1^ per mmol l^−1^, 95% CI 4.6–7.8; *P*<0.0001) (Supplementary Figure S1). EPA alone has no effect (Model 1). Ethyl-EPA increases RBC EPA (+22.5 μg g^−1^, 13.9–31.1; *P*<0.0001) (Supplementary Figure S2a). Vitamins alone have no effect (Model 1). The effects of EPA and Vitamins on RBC PUFA, DHA and ARA are markedly modified by baseline PUFA (Table 3, Supplementary Figures S2b and C). Thus, ethyl-EPA increases PUFA, DHA and ARA strongly in a low PUFA patient, whereas there is no effect in a high PUFA patient. Vitamins have no significant effects on PUFA, DHA and ARA. Adding Vitamins to ethyl-EPA neutralizes the increase in these fatty acids in a low PUFA patient (Model 3A, Supplementary Figures S2b and C), thus the effects of Vitamins-EPA and placebo do not differ (*P*>0.86). In a high PUFA patient, the combined treatment reduces ARA (−41.3 μg g^−1^,–67.9 to −14.7; *P*=0.003) (Model 3B, Supplementary Figures S2B).

Changes in Positive subscale scores, in particular Suspiciousness/persecution (P6), and ARA are positively linked across baseline PUFA (Pearson's *r*=0.27, *P*=0.02; *r*=0.33, *P*=0.005, respectively, *n*=71; Supplementary Figures S3A and B). Changes in positive symptoms and s-alpha-tocopherol are positively linked (*t*=+2.2, *P*=0.03), but only in low PUFA patients (Supplementary Figure S4). The effect of change in alpha-tocopherol remains significant (*t*=+2.1, *P*=0.04) in low PUFA patients after adjusting for change in ARA, which has no effect in this model. Changes in total PANSS and the Negative and General Psychopathology Subscales are not linked to changes in PUFA or alpha-tocopherol. Changes in serum MDA and F2-isoprostane are unrelated to changes in PANSS. The effects on biomarkers of oxidative stress and general health are reported at http://www.nature.com/tp (Supplementary Text, Supplementary Tables S3 and S4). Several effects had p-values between 0.05 and 0.20. This concerns the variables Total PANSS, General Psychopathology Subscale, antipsychotic drug dosage, diastolic and systolic blood pressure, body mass index, MCV, MCHC, platelet count, CRP, potassium, creatinine, albumin, ALAT, g-GT, triglycerides, glucose, free thyroxine, TSH, F2-isoprostane, RBC PUFA, ω-6/ ω-3 ratio, DHA, and ARA.

## Discussion

This is the first placebo-controlled trial of an omega-3 fatty acid and redox regulators for treating schizophrenia. We have shown that adding ethyl-EPA 2 g day^−1^ or vitamin E 364 mg day^−1^+vitamin C 1000 mg/day to antipsychotic drugs impairs the course of psychosis, but only among patients with low levels of RBC PUFAs. Combining EPA and the vitamins neutralizes this detrimental effect. We also observed that patients with low PUFA levels have thrice the risk of drop-out as those with high PUFA levels at baseline. EPA and the vitamins, given separately, increase the risk of drop-out. These effects are neutralized by combining the agents.

A recent meta-analysis of randomized, placebo-controlled trials showed a modest non-significant beneficial effect of fatty acids in schizophrenia (0.24, *P*=0.08, 95% CI −0.03 to 0.51).^7^ In contrast, remarkable effects of EPA-rich oils have been found for psychosis prevention and depression.^8,23^ Psychotogenic effects have not been reported in previous EPA trials at doses of 1–4 g day^−1^. Why do results in our study differ from others? The phase of the disorder may have an impact on outcome. In contrast to most trials,^7^ ours was run during a psychotic exacerbation, when there is evidence of increased oxidative stress.^6^ This can modify the effects of the trial agents. Patients eating more seafood, as in Norway, will probably benefit less from added EPA. A daily intake of 3–6 g of EPA-DHA is generally considered safe.^24^ It is conceivable, however, that a diet rich in long-chain omega-3 fatty acids may increase the risk that 2 g of EPA induce adverse effects in certain patients with schizophrenia. Finally, some of the discrepancies may be due to differing statistical methods. These may explain why we detected worsening possibly ignored in previous trials. We used linear mixed models, which are particularly well suited for analyzing unbalanced longitudinal data.^15,16^ We included PUFA baseline levels in the models predicting outcome, which has not been done in previous trials. This improved the models' fitness to the data immensely. It appears especially important to take baseline PUFA into account as an effect modifier, as recognized in a recent review.^7^

High doses of vitamin E against tardive dyskinesia have yielded neither anti- nor pro-psychotic effects.^9^ The only randomized controlled trial of vitamin C with adequate sample size shows a highly significant beneficial effect.^10^ Other randomized trials with redox regulators in schizophrenia are few but promising.^11,12^ The two open-label trials with a combination of omega-3 fatty acids and vitamins E and C in a chronic phase suggest clinical relevant effects.^25,26^ Possibly, a beneficial effect is revealed only during a stabilized phase of the disorder.

Why do the trial drugs worsen psychosis? The most likely mechanism is by increasing oxidative stress. Oxidative stress disturbs functioning of neurons and neuroglia,^5^ inhibits NMDA-receptors^27^ and impairs dopamine modulation,^28^ yielding psychotic symptoms despite antipsychotic medication. Several defense systems against oxidative stress have been shown to be abnormal in schizophrenia. Among these abnormalities the best validated seems to be an endogenous glutathione deficiency.^5,29, 30, 31, 32, 33^ However, the deficient redox regulation might be limited to a subgroup of the patients, who may be characterized by low levels of RBC PUFAs during an acute episode.^6,13^ This is in keeping with the results of the present trial. Adding ethyl-EPA 2 g d^−1^ causes a marked increase in long-chain PUFA, which in this environment could be peroxidized.^34^ Similarly, vitamin E at high doses behaves as a pro-oxidant in a milieu with insufficient antioxidant capacity.^35,36^ It is unlikely that vitamin C at high doses causes the psychotogenic effect.^10,37^ In the present study, the combination of vitamins E and C was expected to yield a better clinical effect than giving each vitamin separately.^38^ Particularly, ascorbic acid reduces the α-tocopheryl radical back to α-tocopherol, counteracting a pro-oxidant effect of vitamin E. The synergistic effect of vitamins E+C and EPA suggests mutual redox regulation. Thus, the vitamins may reduce oxidized PUFA,^34^ and EPA may reduce oxidized vitamins E+C.^39^ The resulting neutral redox regulatory effect is reflected in a neutral effect on the course of psychosis.

Why does baseline PUFA modify the effects of both EPA and Vitamins E+C in our study? Low serum levels of α-tocopherol were the strongest predictor of low PUFA levels at baseline.^6^ This indicates a specific redox-regulatory problem which will influence the effects of both EPA and Vitamins. Overall, it seems to interact more strongly with Vitamins. Possibly, in low PUFA patients, α-tocopherol at these doses is more likely to behave as a pro-oxidant than EPA, implying a stronger psychotogenic effect. This is reflected in an increased antipsychotic dosage for Vitamins, but not for EPA. Accordingly, baseline PUFA modifies highly significantly the effect of vitamins on blood pressure in our study, although there is no significant interaction with EPA (Table 2, Supplementary Text). It is well known that oxidative stress raises blood pressure, partly by cerebral regulation.^40, 41, 42^ There is evidence from rodent experiments and clinical trials that vitamins E and C may either reduce or increase blood pressure.^43, 44, 45^ In our study, EPA causes a non-significant increase in systolic blood pressure in the low PUFA patients only, in contrast to the antihypertensive effect found in several clinical studies.^46,47^ This suggests that oxidative stress may change the direction of effect of EPA on blood pressure, but to a modest degree.

Other mechanisms might contribute to the effects of study drugs. The psychotogenic effect of EPA could be mediated by endocannabinoids, derived from arachidonic acid.^48,49^ In our trial, EPA treatment entails increased RBC arachidonic acid, but only at low baseline PUFA levels and only when EPA is not combined with vitamins E+C. An increase in RBC arachidonic acid is linked to increased positive symptoms, unopposed by presumably adequate antipsychotic medication. Accordingly, antidopaminergic drugs are ineffective against cannabinoid CB_1_ receptor agonists.^50^ In our study, increases in RBC long-chain PUFA are linked to worsening of symptoms, whereas other trials have shown the opposite.^51,52^ Either these fatty acids do not cause the worsening, and they may thus be considered a confounding factor, or the impact fatty acids, especially arachidonic acid, have on symptoms is modified by other factors.

Vitamin E's psychotogenic effect could be due to the displacement of beneficial γ- and δ-tocopherols by high doses of α-tocopherol.^53,54^ Also, too high concentrations of α-tocopherol could disturb its regulation of enzyme and gene activities related to cell signaling and membrane processes.^55^ However, it is not obvious how such effects of α-tocopherol could be modified by PUFA levels or the combination with EPA. Thus, it is less likely that these mechanisms account for our findings.

Probably, pharmacokinetic interactions between trial agents and antipsychotic drugs, mediated by the activities of cytochrome P450 enzymes^56, 57, 58^ and p-glycoprotein,^59^ do not explain the increase in psychosis. Types and doses of antipsychotics and compliance with antipsychotic medication did not differ between treatment groups and are therefore unrelated to our results.

We cannot explain why low PUFA patients are more prone to drop out, independently of adverse events and trial drugs. We assumed that negative symptoms could be a mediator, because they entail less ability to comply with the trial, and because they are linked to low PUFA at baseline,^13,60^ but this hypothesis was not verified.

Sensitivity analyses support the validity of all major results. Particularly, application of bootstrapping indicates that these findings are not spurious or due to selection bias. In general, the results are more significant in the PP group, despite a smaller sample size. Worst case analysis indicates that it is highly unlikely that EPA or vitamins would be beneficial in this group of patients, regardless the missing values for drop-out patients. Consistency across different outcome variables supports our main conclusions. Similar patterns emerge for the prediction of blood pressure, RBC fatty acid levels, and drop-out as for positive symptoms. Use of vitamins entails a raise in antipsychotic dosage at lower PUFA levels, probably as a response to increased psychosis.

The impact of study drugs on the main outcome measures is probably underestimated. This concerns especially the beneficial effect of adding vitamins E+C to EPA. Group 4 was particularly small by chance, because a simple randomization procedure was applied instead of the planned block randomization. The discrepancy between planned and obtained sample size, due to recruitment problems, entails a reduced statistical power. It is likely that several effects that had p-values between 0.05 and 0.20 would become statistically significant if the sample size was doubled. Thus, the impact of these dietary agents, especially in the low PUFA patients, is probably much broader than shown in the present trial because of insufficient sample size. The multicenter design, entailing larger assessment variance, also reduced the statistical power. Study characteristics *increasing* the statistical power include a homogenous population (phase of disorder, ethnicity), a high completer rate, extensive training of investigators and efficient statistical procedures.

Generalizations should be done cautiously. We do not know the effects of the trial drugs in patients without antipsychotics, at disease onset, during a stabilized phase of the disorder, or on a different diet. Lower doses of agents could be better tolerated.^25,38^ Combinations of omega-3 fatty acids with other antioxidants could be more efficient.^11,12,54^ These issues should be explored in future trials.

In conclusion, during an acute episode, patients with schizophrenia do not benefit from additional EPA and vitamins E and C. Unexpectedly, these dietary substances, given separately at moderately high doses, induce psychotic symptoms in patients with low blood levels of PUFA. However, combined, these agents seem safe.

## Figures and Tables

**Table 1:** Sample characteristics at baseline

*Characteristics*	*Groups in study*			
	*Placebo EPA+placebo vitamins, Group 1*	*Placebo EPA+active vitamins, Group 2*	*Active EPA+placebo vitamins, Group 3*	*Active EPA+active vitamins, Group 4*
Age, mean (s.d.) (years)	28.3 (5.8)	28.6 (6.3)	25.7 (5.4)	27.6 (7.1)
Male, *n* (%)	17 (71)	14 (54)	20 (61)	12 (64)
Education, % ((⩽1):2:3))^a^	17:50:33	27:50:23	39:52:9	31:56:13
Smoker, *n* (%)	14 (58)	19 (73)	20 (61)	10 (63)
Diagnosis, *n* (SZ:SA:SF)^b^	21:2:1	16:9:1	21:7:5	13:2:1
Duration of illness, median (25–75%), year	7 (2–10)	3.5 (2–8)	2 (1–5.5)	3.5 (1–6.5)
First hospitalization, *n* (%)	7 (29)	5 (19)	14 (42)	51(31)
Clozapine or olanzapine, *n* (%)	12 (50)	15 (58)	16 (49)	8 (50)
DDD antipsych., median (interquartile range)^c^	1.9 (0.6)	1.7 (1.0)	1.1 (1.0)	1.2 (0.6)
Total PANSS^d^	82.5 (19)	78.5 (30)	78 (16)	94.5 (30)
Positive subscale	20 (6)	21 (9)	18 (8)	23.5 (7)
Negative subscale	19.5 (12)	19.5 (14)	21 (11)	26 (8)
General psychopathology subscale	42 (10)	37 (15)	37 (10)	46.5 (13)
RBC PUFA^e^	341 (335)	275 (421)	405 (304)	426 (275)
EPA	7.3 (9.4)	6.6 (9.7)	9.0 (10.6)	9.9 (10.5)
DHA	37 (53)	32 (59)	48 (54)	54 (44)
ARA	110 (115)	81 (144)	117 (122)	117 (94)
Lipid-adjusted s-alpha-tocopherol^f^	4.3 (2.5)	4.9 (4.8)	4.9 (3.6)	4.9 (2.4)

Abbreviations: ARA, Arachidonic acid; DDD, defined daily doses; DHA, Docosahexaenic acid; EPA, eicosapentaenoic acid; PANSS, Positive and Negative Syndrome Scale; PUFA, polyunsaturated fatty acid; RBC, red blood cells. Sample sizes of patients differed according to whether the specific assessment was performed. ITT sample: *n*_ITT_=24, 26, 33 and 16 for Groups 1–4, respectively. Below *n*=*n*_ITT_ if not otherwise specified.

a Highest completed education:≤1 =primary school completed or non-completed, 2=secondary school, 3 =college or university.

b Diagnoses (DSM-IV): SZ, schizophrenia; SA, schizoaffective disorder; SF, schizophreniform disorder.

c Defined daily doses of prescribed antipsychotic drugs.

d PANSS, Total sum and the three a priori subscales.

e RBC PUFA = sum of ω-3 and ω-6 PUFAs in red blood cells, μg g^−1^ RBC. For all fatty acids derived variables: *n=*24, 24, 33 and 16.

f s-alpha-tocopherol/(triglyceridescholesterol), (μmoll^−1^)/(mmo11^−1^), *n*=22, 24, 31 and 14. ^c-f^Median (interquartile range).

**Table 2:** Estimated changes from baseline to week 16: symptoms, functioning and vital signs

*Outcome variable*	*Low PUFA patient*					*High PUFA patient*					*Treatment* × *Time* × *PUFA*		
	*β*_*0*_	*β*_*1*_	*β*_*2*_	*β*_*3*_	*β*_*4*_	*β*_*0*_	*β*_*1*_	*β*_*2*_	*β*_*3*_	*β*_*4*_	*V* × *T* × *P*	*E* × *T* × *P*	*V* × *E* × *T* × *P*
Total PANSS	80.8 (74.9 to 86.7)	−22.4 (−28.7 to −16.0) <0.0001	9.2 (−2.6 to 21.0) 0.12	12.6 (1.0 to 24.4) 0.03	−22.8 (−49.0 to 3.4)0.09	81.0 (76.7 to 85.2)	−22.4 (−28.7 to −16.0)<0.0001	0.8 (−9.0 to 10.6) 0.98	−0.08 (−8.8 to 8.6) 0.87	0.2 (−14.0 to 14.4) 0.98	0.12	0.02	0.09
Positive subscale – PANSS	18.7 (17.0 to 20.4)	−7.7 (−9.7 to −5.6) <0.0001	5.4 (1.6 to 9.0) 0.005	5.6 (2.0 to 9.4) 0.003	−10.0 (−18.4 to −1.8) 0.02	20.1 (18.9 to 21.3)	−7.7 (−9.7 to −5.6) <0.0001	−0.4 (−3.5 to 2.6) 0.77	1.3 (−1.4 to 4.1) 0.34	1.1 (−3.4 to 5.6) 0.63	0.001	0.01	0.008
Negative subscale – PANSS	22.6 (20.4 to 24.9)	−3.6 (−5.8 to −1.5) <0.0001	−1.8 (−5.8 to 2.1) 0.35	0.7 (−3.2 to 2.7) 0.71	−2.6 (−11.5 to 6.2) 0.55	20.6 (19.0 to 22.2)	−3.6 (−5.8 to −1.5) <0.0001	−0.4 (−3.7 to 2.8) 0.79	−1.9 (−4.8 to 1.0) 0.20	0.1 (−4.6 to 4.9) 0.96	0.42	0.16	0.53
General psychopathology subscale – PANSS	39.5 (36.6 to 42.4)	−10.8 (−14.1 to −7.5) <0.0001	5.2 (−0.9 to 5.7) 0.095	5.7 (−0.4 to 10.8) 0.07	−10.4 (−24.1 to 3.2) 0.13	40.2 (38.1 to 42.3)	−10.8 (−14.1 to −7.5) <0.0001	1.5 (−3.6 to 6.5) 0.56	0.04 (−4.5 to 5.5) 0.99	−0.5 (−8.0 to 6.9) 0.90	0.19	0.052	0.15
Severity of adverse events – UKU	2.4 (1.5 to 3.3)	−0.6 (−1.6 to 0.6) 0.32	0.1 (−1.8 to 2.0) 0.89	−1.0 (−3.0 to 0.8) 0.27	0.04 (−4.2 to 4.2) 0.98	2.2 (1.5 to 2.8)	−0.6 (−1.6 to 0.6) 0.32	0.4 (−1.2 to 2.0) 0.67	−0.2 (−1.6 to 0.6) 0.67	0.1 (−2.2 to 2.4) 0.95	0.81	0.41	0.99
Antipsychotic drugs – DDD	1.4 (1.1 to 1.6)	−0.1 (−0.4 to 1.4) 0.41	0.4 (−0.1 to 0.8) 0.10	0.1(−0.4 to 0.6) 0.80	−0.4 (−1.4 to 0.6) 0.39	1.7 (1.5 to 1.9)	−0.1 (−0.4 to 1.4) 0.41	−0.2 (−0.6 to 0.2) 0.45	0.1(−0.2 to 0.4) 0.66	0.02 (−0.4 to 0.8) 0.58	0.01	0.94	0.24
Blood pressure, diastolic, mm Hg	75.6 (72.7 to 78.4)	2.8 (−1.0 to 6.7) 0.15	4.8 (−2.4 to 11.9) 0.19	1.4 (−5.6 to 8.5) 0.69	−12.8 (−28.9 to 3.2) 0.12	77.2 (75.0 to 79.3)	2.8 (−1.0 to 6.7) 0.15	−3.8 (−9.5 to 2.0) 0.20	−1.6 (−6.9 to 3.6) 0.54	0.2 (−8.4 to 8.8) 0.97	0.01	0.37	0.11
Blood pressure, systolic	119.7 (115.8 to 123.6)	−0.7 (−6.0 to 4.7) 0.80	9.2 (−0.6 to 19.0) 0.07	8.1 (−1.6 to 17.9) 0.10	−15.9 (−38.0 to 6.1) 0.16	122.9 (120.0 to 125.8)	−0.7 (−6.0 to 4.7) 0.80	−4.4 (−12.2 to 3.5) 0.28	1.0 (−6.2 to 8.3) 0.78	1.9 (10.0 to 13.7) 0.75	0.003	0.14	0.12

The linear mixed models (Model 2) have been centered on the means of red blood cells PUFA in the low (=102 μg g^−1^ RBC; Model 3A) and high (=441 μg g^−1^ RBC; Model 3B) PUFA groups, respectively. Effect coefficients: *β*_0_ Intercept (estimated baseline value), *β*_1_ Time, *β*_2_ Time × Vitamins, *β*_3_ Time × EPA, *β*_4_ Time × Vitamins × EPA. Time: 0 weeks, 0; 16 weeks, 1. EPA: EPA-placebo, 0; EPA-active, 1; Vitamins: Vitamins-placebo, 0; Vitamins-active, 1. For each effect, the estimate, the 95% confidence interval and the *p*-value (except β0) are reported. In the Treatment × Time × PUFA columns, the *p*-values of interaction effects with PUFA are displayed (V × T × P Vitamins × Time × PUFA, E × T × P EPA × Time × PUFA, V × E × T × P Vitamins × EPA × Time × PUFA). NB! PUFA is treated as a continuous (not low versus high) variable in these models. *P*-values are in red if <0.05, in dark red if 0.05<*P*-value<0.10. PUFA, polyunsaturated fatty acids; PANSS, Positive and Negative Syndrome Scale; UKU, UKU Side Effect Rating Scale; DDD, Defined Daily Doses.

**Table 3:** Estimated changes from baseline to week 16: Fatty acids and alpha-tocopherol

*Outcome variable*	*Low PUFA patient*					*High PUFA patient*					*Treatment × Time × PUFA*		
	*β*_*0*_	*β*_*1*_	*β*_*2*_	*β*_*3*_	*β*_*4*_	*β*_*0*_	*β*_*1*_	*β*_*2*_	*β*_*3*_	*β*_*4*_	*V × T × P*	*E × T × P*	*V × E × T × P*
RBC PUFA^a^	110.6 (80.7–140.6)	39.2 (−7.9–86.3) 0.10	66.3 (−14.8–147.5) 0.11	229.9 (147.9–312.0) <0.0001	−302.1 (−485.2–−119.0) 0.001	437.2 (415.4–459.1)	39.2 (−7.9–86.3) 0.10	−52.0 (−120.5–16.4) 0.14	19.8 (−39.7–79.4) 0.51	−10.2 (−110.7–90.3) 0.84	0.003	<0.0001	0.002
EPA^a^	2.1 (−1.8–6.1)	5.1 (−1.1–11.3) 0.11	3.7 (−7.0–14.4) 0.50	24.2 (13.4–35.0) <0.0001	−28.6 (−52.7–4.4) 0.02	11.7 (8.8–14.6)	5.1 (−1.1–11.3) 0.11	−0.4 (−9.4–8.7) 0.94	21.0 (13.2–28.8) <0.0001	5.3 (−8.0–18.5) 0.43	0.43	0.55	0.006
DHA^a^	9.4 (3.1–15.6)	6.3 (−2.5–15.1) 0.16	15.4 (−0.2–30.9) 0.053	30.6 (14.8–46.3) <0.0001	−48.5 (−84.1–13.0) 0.008	55.7 (51.2–60.2)	6.3 (−2.5–15.1) 0.16	−6.1 (−19.4–7.1) 0.36	−1.1 (−12.6–10.4) 0.86	−5.4 (−24.9–14.2) 0.59	0.004	<0.0001	0.02
ARA^a^	24.9 (14.3–35.5)	13.3 (−3.0–29.5) 0.11	19.4 (−8.9–47.7) 0.18	65.0 (36.4–93.6) <0.0001	−87.1 (−151.1–−23.1) 0.008	136.8 (129.1–144.5)	13.3 (−3.0–29.5) 0.11	−18.7 (−42.6–5.2) 0.12	−9.0 (−29.7–11.8) 0.40	−13.6 (−48.7–21.5) 0.45	0.005	<0.0001	0.03
S-alpha-tocopherol (lipid adjusted)^b^	4.2 (3.3–5.0)	−0.2 (−1.3–0.9) 0.67	4.9 (2.9–6.9) <0.0001	0.9 (−1.0–2.8) 0.35	−1.6 (−6.1–2.8) 0.47	6.1 (5.5–6.7)	−0.2 (−1.3–0.9) 0.67	6.3 (4.6–8.0) <0.0001	0.5 (−1.0–2.0) 0.47	−0.1 (−2.6–2.4) 0.93	0.16	0.71	0.50

Abbreviations: ARA, arachidonic acid; DHA, docosahexaenoic acis; EPA, eicosapentaenoic acid; PUFA, polyunsaturated fatty acid; RBC, red blood cell. As for explanation of the linear mixed models, see footnote for Table 2.

a Fatty acids: μg g^−1^

b s-alpha-tocopherol/(triglycerides-cholesterol), (μmoll^−1^)/(mmoll^−1^).
