# Supplementary material for: A randomized placebo-controlled trial of an omega-3 fatty acid and vitamins E+C in schizophrenia
Source: Transl Psychiatry. 2013 Dec 17;3(12):e335–. doi: 10.1038/tp.2013.110 (PMC3906471; doi:10.1038/tp.2013.110)
Supplement: Supplementary information [file tp2013110x1.doc]

**2013TP000138R**

**Revised version 1**

**A RANDOMISED PLACEBO-CONTROLLED TRIAL OF AN OMEGA-3 FATTY ACID AND VITAMINS E + C**

**IN SCHIZOPHRENIA**

**SUPPLEMENTARY MATERIAL - TEXT**

**www.nature.com/tp**

Håvard Bentsen, M.D., Ph.D.^1,2^, Kåre Osnes, M.D., Ph.D.^3^ , Helge Refsum, M.D., Ph.D.^1^, Dag K. Solberg, M.D.^1^, Thomas Bøhmer, M.D., Ph.D.^4^

^1^Center for Psychopharmacology, Diakonhjemmet Hospital, 0319 Oslo, Norway; ^2^Division of Psychiatry, Oslo University Hospital, Aker, 0514 Oslo, Norway; ^3^Department of Psychosomatic Medicine, Oslo University Hospital, Rikshospitalet, 0424 Oslo, Norway;  ^4^Nutritional Laboratory, Dept. of Medical Biochemistry, Oslo University Hospital, Aker, 0424 Oslo, Norway.

*Corresponding author:* Dr. Håvard Bentsen, Center for Psychopharmacology, Diakonhjemmet Hospital, P.o.b. 85, Vinderen, 0319 Oslo, Norway. Phone +47 22454662/ 22029940; 48007446. Fax +47 22454698. [havard.bentsen@medisin.uio.no](mailto:havard.bentsen@medisin.uio.no)

**Assessment methods^1,2^**

Patients were assessed from the start to the end of the trial at five visits with four weeks intervals. Observers and patients were blind to PUFA grouping.

The main clinical instrument was the Positive, Negative and General psychopathology Scale (PANSS) - Structured Interview Version, applied at start, after 8 and 16 weeks (Multi-Health Systems Inc., Toronto, ON, Canada M2H 3M6). The 30 items rated from 1 (best) to 7 (worst) were grouped according to the Positive, Negative and General psychopathology subscales.^3^ The inter-rater reliability in our group of 16 investigators was assessed by rating of ten (median) videotaped interviews before and during the trial.^4^ The inter-rater reliability coefficients [ICC (1,1)] were 0.73 (95% c.i. 0.55-0.90) for Total PANSS, 0.74 (0.56-0.90) for Positive subscale, 0.78 (0.62-0.92) for Negative subscale, and 0.62 (0.41-0.84) for General psychopathology subscales.

Global Assessment of Functioning was assessed by the Split Version (S-GAF). Symptom Scale and Function Scale, both with ratings from 0 (worst) to 100 (best).^5^ The ICC (1,1) were S-GAF-S 0.82 (0.71-0.91) and S-GAF-F 0.77 (0.64-0.87). Adverse effects were assessed at each visit with the UKU Side Effect Rating Scale (USERS), a comprehensive four levels rating scale for psychotropic drugs.^6^ Eight items only, regarding parkinsonism, akathisia, impaired haemostasis, gastrointestinal adverse effects and sedation, were systematically screened for. Other adverse effects were asked for by one open question (“Have you otherwise had trouble or noticed body changes the last month?”), coded and rated according to USERS. Concomitant medication was classified according to The Anatomical Therapeutic Chemical Classification System (ATC) and doses measured as Defined Daily Doses (ATC/DDD).

We assessed vital signs (weight, height, blood pressure, heart rate) and analysed blood samples at baseline and at the end of the trial. Fasting cholesterol, triglycerides and glucose were analysed at the Department of Clinical Chemistry, Aker University Hospital with enzymatic methods from Roche Diagnostics Norge AS, Oslo, Norway. Other ordinary biochemical analyses of serum or blood were performed at the patient’s hospital.

Blood for fatty acid analyses was sampled from the patient after an overnight fast. For analysis of fatty acids, EDTA-blood was kept cool until centrifugation within one hour. Washed red blood cells were stored at -70^o^C within one hour after centrifugation and sent within three months in dry ice to Mylnefield Research Services Ltd., Dundee, U.K. There lipids were extracted, converted into fatty acid methyl esters and analysed by gas chromatography, yielding fatty acid profiles. Twenty-eight species of fatty acids from C14:0 to C24:1 were reported as µg/g RBC. The sum of ω3 fatty acids was C:18:3(n-3)+C:18:4(n-3)+C:20:3(n-3)+C:20:5(n-3)+C:22:5(n-3)+C:22:6(n-3). The sum of ω6 fatty acids was C:18:2(n-6)+C:18:3(n-6)+C:20:2(n-6)+C:20:3(n-6)+C:20:4(n-6)+C:22:4(n-6)+C:22:5(n-6). The sum of ω3 and ω6 fatty acids was named Polyunsaturated Fatty Acids (PUFA), the key variable of the present study. The sum of ω3 and ω6 PUFA with 20 or 22 carbon atoms was named Long chain Polyunsaturated Fatty Acids (LCPUFA).

Serum α-tocopherol was analysed at the Nutrition Laboratory, Oslo University Hospital, Aker (OUHA) with Bio-Rad kits, High Performance Liquid Chromatography (HPLC) and Ultraviolet light (UV) detection. The adjusted term [α-tocopherol]/([triglycerides] + [cholesterol]) was used in statistical analyses. Serum Total Antioxidant Status (TAS) was analysed at the Nutrition Laboratory, OUHA with Randox kits. Total serum malondialdehyde (MDA) was analysed with the colorimetric thiobarbituric acid reactive substances (TBARS) test. A coefficient of variation of 6.5 % was achieved. Analyses were done by Vitas AS, Oslo. For analysis of free F2-isoprostane (8-epi-PGF_2α_ or iPF_2α_-III), EDTA-blood was kept cool until centrifugation within one hour. Plasma was stored at -70^o^C within one hour after centrifugation. After purification on a F2-isoprostane affinity column, the plasma levels were quantified with competitive enzyme-linked immunoassay followed by UV detection at 405-420 nm (Cayman Chemical 516351 EIA kit). Analyses were done by the Hormone Laboratory, OUHA.

**Effects of Study Drugs on Biomarkers of General Health**

We explored the effects of study drugs and PUFA level on the course of all vital signs and biochemical variables presented in Tables 1-2 of our previous article.^2^ Significant findings from linear mixed models analyses (Models 1-3) are reported below. For complete results, see Tables S2- 3, www.nature.com/tp.

1. **Blood pressure.** Vitamins increase systolic blood pressure (p=0.03), but this effect depends strongly on the PUFA level (interaction term: <0, p=0.003) (Model 2). Thus, it is found only in the low PUFA patient (Cohen’s *d* =0.32; p=0.07) (Model 3A, Table 2). Vitamins increase non-significantly diastolic blood pressure (p=0.09). Again, the effect is modified by PUFA (<0, p=0.01). In the low PUFA patient, combining EPA and Vitamins prevents non-significantly these effects of study drugs on blood pressure.
2. **Mean corpuscular haemoglobin concentration (MCHC).** Across PUFA, Vitamins increase MCHC (+0.7; 95% CI, 0.1 - 1.3; *d* =0.43; p=0.02) (Model 1). Thus, similar increases are found in the low as in the high PUFA patients (Table S4). Adding Vitamins+EPA increases MCHC compared to double placebo (+2.5; 95% CI, 0.4 - 4.6; Cohen’s *d*=0.49; p=0.02) (Model 1). PUFA at baseline is a strong negative predictor of MCHC during the trial (Model 2: p=0.00008).^1^
3. **Potassium.** Vitamins reduce potassium (p=0.03), but this effect depends on the PUFA level (interaction term: >0, p=0.01) (Model 2). Thus, it is found only in the low PUFA patient (*d* =0.29; p=0.07) (Model 3A, Table 2).
4. **Creatinine.** Across PUFA, Vitamins reduce creatinine (-5.3; 95% CI, -10.2 - -0.4; *d* = 0.45; p=0.03), EPA tends to reduce it (-3.5; 95% CI, -8.0 - 1.0; p=0.13), whereas adding EPA to Vitamins counteracts these effects (+7.9; 95% CI, 0.5 - 15.2; p=0.04) (Model 1). The effect of Vitamins+EPA does not differ from double placebo. Similar effects are found in low as in high PUFA patients (Table S4).
5. **Uric acid.** EPA increases uric acid (p=0.047) (Model 2). There is a trend that PUFA is a negative effect modifier (p=0.10). Thus, the increase is found only in the low PUFA patient (*d=*0.42; p=0.048) (Model 3A, Table S4).
6. **Cholesterol.** Across PUFA, combining EPA and Vitamins counteracts the non-significant increasing effects of each of them (Model 1; interaction Vitamins*EPA: <0, p=0.02). The effect of adding EPA to Vitamins depends strongly on the PUFA level (<0, p=0.008) (Model 2, Table S4). In a high PUFA patient, Vitamins alone increase cholesterol (*d* =0.42; p=0.04), which is strongly counteracted by adding EPA (p=0.001) (Model 3B, Table S4). Replacing Vitamins*EPA by Vitamins+EPA in Model 3B, thus comparing the combined agents to double placebo, yielded a trend effect (p=0.11) of Vitamins+EPA on s-cholesterol in a high PUFA patient.
7. **Glucose.** EPA tends to reduce glucose (p=0.06), however there is an interaction with PUFA (>0, p=0.03) (Model 2). Thus, there is a trend only in the low PUFA patient, that EPA entails a decrease in glucose during the trial (<0, p=0.10).

Summarizing, in the low PUFA patient only, Vitamins tend to increase the systolic blood pressure and reduce potassium, and EPA increases uric acid and tends to reduce glucose. In the high PUFA patient only, Vitamins increase cholesterol and the combination of Vitamins and EPA tends to it. Across PUFA, Vitamins increase MCHC and reduce creatinine.

References:

1. Bentsen H, Solberg DK, Refsum H, Gran JM, Bøhmer T, Torjussen PA *et al.* Bimodal distribution of polyunsaturated fatty acids in schizophrenia suggests two endophenotypes of the disorder. *Biol Psychiatry* 2011; **70**: 97-105
2. Bentsen H, Solberg DK, Refsum H, Bøhmer T. Clinical and biochemical validation of two endophenotypes of schizophrenia defined by levels of polyunsaturated fatty acids in red blood cells. *Prostaglandins Leukot Essent Fatty Acids* 2012; **87**: 35-41
3. Kay SR. Positive and Negative Syndromes in Schizophrenia. Assessment and Research. Brunner/Mazel, New York, 1991
4. Bentsen H, Munkvold OG, Notland TH, Boye B, Bjørge H, Lersbryggen AB *et al.* The interrater reliability of the Positive and Negative Syndrome Scale (PANSS). *Int J Methods Psychiatr Res* 1996; **6**: 227-235
5. Pedersen G, Hagtvet KA, Karterud S. Generalizability studies of the Global Assessment of Functioning-Split version. *Compr Psychiatry* 2007; **48**: 88-94
6. Lingjærde O, Ahlfors UG, Bech P, Dencker SJ, Elgen K. The UKU side effect rating scale. A new comprehensive rating scale for psychotropic drugs and a cross-sectional study of side effects in neuroleptic-treated patients. *Acta Psychiatr Scand* 1987; **76**: 1-100

**A RANDOMISED PLACEBO-CONTROLLED TRIAL OF AN OMEGA-3 FATTY ACID AND VITAMINS E + C**

**IN SCHIZOPHRENIA**

**Bentsen H *et al***

**SUPPLEMENTARY MATERIAL – TABLES**

**www.nature.com/tp**

**Table S1: Sample characteristics at baseline – Treatment groups**

| **Characteristics** | **Placebo EPA +**  **Placebo vitamins**  *Group 1* | **Placebo EPA +**  **Active vitamins** *Group 2* | **Active EPA +**  **Placebo vitamins** *Group 3* | **Active EPA +**  **Active vitamins**  *Group 4* |
| --- | --- | --- | --- | --- |
| Age, *mean (SD), y* | 28.3 (5.8) | 28.6 (6.3) | 25.7 (5.4) | 27.6 (7.1) |
| Male, *n (%)* | 17 (71) | 14 (54) | 20 (61) | 12 (64) |
| Education, % *[(≤ 1):2:3)]* ^1^ | 17:50:33 | 27:50:23 | 39:52:9 | 31:56:13 |
| Smoker*, n (%)* | 14 (58) | 19 (73) | 20 (61) | 10 (63) |
| Diagnosis, *n* *(SZ:SA:SF)* ^2^ | 21:2:1 | 16:9:1 | 21:7:5 | 13:2:1 |
| Duration of illness, *median (25-75 perc*.*), y* | 7 (2-10) | 3.5 (2-8) | 2 (1-5.5) | 3.5 (1-6.5) |
| First hospitalization, *n (%)* | 7 (29) | 5 (19) | 14 (42) | 51 (31) |
| Clozapine or olanzapine, *n (%)* | 12 (50) | 15 (58) | 16 (49) | 8 (50) |
| DDD antipsych.,  *median (interquartile range)* ^3^ | 1.9 (0.6) | 1.7 (1.0) | 1.1 (1.0) | 1.2 (0.6) |
| GAF-S ^4^ | 32 (5) | 36 (9) | 36 (9) | 36 (12) |
| GAF-F | 34 (8) | 38 (6) | 35 (7) | 33 (11) |
| Total PANSS ^5^ | 82.5 (19) | 78.5 (30) | 78 (16) | 94.5 (30) |
| Positive Subscale | 20 (6) | 21 (9) | 18 (8) | 23.5 (7) |
| Negative Subscale | 19.5 (12) | 19.5 (14) | 21 (11) | 26 (8) |
| General Psychopathology Subscale | 42 (10) | 37 (15) | 37 (10) | 46.5 (13) |
|  | | | | |
| **Characteristics (ctd.)** | **Placebo EPA +**  **Placebo vitamins**  *Group 1* | **Placebo EPA +**  **Active vitamins** *Group 2* | **Active EPA +**  **Placebo vitamins** *Group 3* | **Active EPA +**  **Active vitamins**  *Group 4* |
| Rbc PUFA ^6^ | 341 (335) | 275 (421) | 405 (304) | 426 (275) |
| ω-3 PUFA | 73 (86) | 54 (97) | 90 (90) | 99 (72) |
| ω-6 PUFA | 265 (243) | 221 (327) | 305 (218) | 317 (196) |
| ω-6/ ω-3 ratio | 4.0 (3.0) | 4.3 (2.0) | 3.9 (1.7) | 3.6 (1.7) |
| Eicosapentaenoic acid (EPA) | 7.3 (9.4) | 6.6 (9.7) | 9.0 (10.6) | 9.9 (10.5) |
| Docosahexaenoic acid (DHA) | 37 (53) | 32 (59) | 48 (54) | 54 (44) |
| Arachidonic acid (ARA) | 110 (115) | 81 (144) | 117 (122) | 117 (94) |
| Lipid-adjusted s-alpha-tocopherol ^7^ | 4.3 (2.5) | 4.9 (4.8) | 4.9 (3.6) | 4*.*9 (2.4) |
| S-uric acid ^8^ | 315 (140) | 277 (151) | 316 (160) | 327 (66) |
| S-albumin ^9^ | 44 (6) | 42 (6) | 41 (7) | 42 (8) |
| Total antioxidant capacity ^10^ | 1.3 (0.2) | 1.3 (0.2) | 1.3 (0.2) | 1.3 (0.2) |
| F2-isoprostane ^11^ | 35 (16) | 26 (24) | 26 (15) | 31 (41) |
| Malondialdehyde (TBARS) ^12^ | 0.43 (0.16) | 0.46 (0.23) | 0.40 (0.29) | 0.39 (0.16) |

**Footnote Table S1**

^1^ Highest completed education: ≤ 1=primary school completed or non-completed, 2=secondary school, 3=college or university.

^2^ Diagnoses (DSM-IV): SZ=schizophrenia, SA=schizoaffective disorder, SF= schizophreniform disorder.

^3^ Defined Daily Doses of prescribed antipsychotic drugs

Sample sizes of patients differed according - whether the specific assessment was performed. Intent-to-treat (ITT) sample: n_ITT_=24,26,33,16 for *Groups* *1-4,* respectively. Below n=n_ITT_ if not otherwise specified

^4^ Global Assessment of Functioning – Symptom (S) and Function (F) Scales

^5^ Positive and Negative Syndrome Scale , Total sum and the three *a priori* subscales.

^6^ Rbc PUFA = sum of ω-3 and ω-6 polyunsaturated fatty acids in red blood cells, µg/g rbc. For all fatty acids derived variables: n=24,24,33,16

^7^ s-alpha-tocopherol /(triglycerides+cholesterol), (µmol/L)/(mmol/L), n=22,24,31,14

^8^ µmol/L, n=16,18,23,15

^9^ g/L, n=22,25,30,15

^10^ mmol/L, n=24,26,32,15

^11^ pg/mL, n=22,26,32,15

^12^ µmol/L, n=22,24,32,15

^3-12^ median (interquartile range)

**Table S2: Sample characteristics at baseline – PUFA groups**

| **Characteristics** | | **ITT sample** *(n=99 or ^5-16^)* | **Low PUFA** *(n=30 or ^5-16^)* | **High PUFA** *(n=67 or ^5-16^)* |
| --- | --- | --- | --- | --- |
| Age, *mean (SD), y* | | 27.4 (6.1) | 29.9 (6.5) ^*^ | 26.3 (5.4) |
| Male, *n (%)* | | 61 (64) | 18 (60) | 43 (64) |
| Education, % *[(≤ 1):2:3)]* ^1^ | | 29:52:19 | 27:50:23 | 30:52:18 |
| Smoker*, n (%)* | | 63 (64) | 19 (63) | 42 (63) |
| Diagnosis, *n* *(SZ:SA:SF)* ^2^ | | 71:20:8 | 23:7:0 | 47:12:8 |
| Duration of illness, *median (interquart. range), y* ^3^ | | 4 (1-9) | 5 (2-10) | 3 (1-7) |
| First hospitalization, *n (%)* | | 31 (31) | 10 (33) | 20 (30) |
| Clozapine or olanzapine, *n (%)* | | 51 (52) | 13 (43) | 36 (54) |
| DDD antipsychotic drugs, ^3, 4^ | | 1.5 (1.0)| | 1.2 (0.8) | 1.5 (1.0) |
| GAF-S ^3^, *^5^* | 35 (10) | | 36 (8) | 35 (9) |
| GAF-F | 35 (8) | | 38 (8) | 35 (7) |
| Total PANSS ^3^, *^6^* | 81 (18) | | 83 (23) | 80 (23) |
| Positive Subscale | 20 (8) | | 21 (9) | 20 (7) |
| Negative Subscale | 21 (12) | | 25 (8) *^*^* | 19 (11) |
| General Psychopathology Subscale | 40 (13) | | 40 (13) | 39 (13) |
| Body Mass Index, *kg/m ^2^* ^3, 7^ | 24.6 (6.4) | | 26.6 (6.9) | 24.2 (5.6) |
| Systolic Blood Pressure, *mean (SD), mm Hg* ^8^ | | 122 (12) | 119 (13) | 123 (12) |
| Diastolic Blood Pressure, *mean (SD), mm Hg* | | 77 (9) | 76 (10) | 77 (8) |
| Cardiac rate, beats per minute, *mean (SD)* ^9^ | | 78 (12) | 79 (14) | 78 (11) |
|  | |  |  |  |
| **Characteristics (ctd)** | | **ITT sample** *(n=99 or ^5-16^)* | **Low PUFA** *(n=30 or ^5-16^)* | **High PUFA** *(n=67 or ^5-16^)* |
| Rbc PUFA, ^3, 10^ | | 384 (343) | 93 (70)^***^ | 460 (148) |
| ω-3 PUFA | | 81 (88) | 11 (11)^***^ | 104 (43) |
| ω-6 PUFA | | 296 (246) | 84 (64)^***^ | 338 (101) |
| ω-6/ ω-3 ratio | | 4.0 (2.0) | 5.8 (2.4)^***^ | 3.6 (1.2) |
| Eicosapentaenoic acid (EPA) | | 8.1 (10.0) | 1.5 (1.4)^***^ | 10.6 (7.2) |
| Docosahexaenoic acid (DHA) | | 45.0 (55.5) | 4.8 (5.3)^***^ | 59.5 (30.4) |
| Arachidonic acid (ARA) | | 114.0 (121.9) | 17.0 (17.9)^***^ | 141.6 (58.6) |
| Lipid-adjusted s-alpha-tocopherol ^3, 11^ | | 4.7 (3.4) | 3.9 (1.2)^***^ | 6.0 (3.6) |
| S-uric acid ^3, 12^ | | 313 (137) | 297 (122) | 321 (139) |
| S-albumin ^3, 13^ | | 42 (7) | 42 (5) | 42 (7) |
| Total antioxidant capacity ^3, 14^ | | 1.3 (0.2) | 1.3 (0.3) | 1.3 (0.2) |
| F2-isoprostane ^3, 15^ | | 29 (20) | 32 (22) | 28 (18) |
| Malondialdehyde (TBARS) ^3, 16^ | | 0.42 (0.21) | 0.39 (0.18) | 0.45 (0.22) |

**Footnote Table S2**

These data have previously been published in: Bentsen H, Solberg DK, Refsum H, Gran JM, Bøhmer T, Torjussen PA *et al.* Bimodal distribution of polyunsaturated fatty acids in schizophrenia suggests two endophenotypes of the disorder. *Biol Psychiatry* 2011; **70**: 97-105. Bentsen H, Solberg DK, Refsum H, Bøhmer T. Clinical and biochemical validation of two endophenotypes of schizophrenia defined by levels of polyunsaturated fatty acids in red blood cells. *Prostaglandins Leukot Essent Fatty Acids* 2012; **87**: 35-41

Sample sizes of patients differed according to whether the specific assessment was performed (notes ^5-16^). Groups were compared with χ^2^ or Fisher’s exact tests, Kendall’s tau-B, or two-sample t-test or Mann-Whitney test of rank differences (^3^), depending on types of data. Low versus high PUFA patients: ^*^ 0.01≤p<0.05, ^**^ 0.001≤p<0.01, ^***^p<0.001.

^1^ Highest completed education: ≤ 1=primary school completed or non-completed, 2=secondary school, 3=college or university.

^2^Diagnoses (DSM-IV): SZ=schizophrenia, SA=schizoaffective disorder, SF= schizophreniform disorder.

^3^Median (interquartile range) is displayed.

^4^ Defined Daily Doses of prescribed antipsychotic drugs

^5^ Global Assessment of Functioning (GAF) – Symptom (S) and Function (F) Scales. ITT n=98, low PUFA n=29, high PUFA n=67

^6^ Positive and Negative Syndrome Scale (PANSS). Possible ranges of scores: Total (30-210 points), Positive (7-49), Negative (7-49 points), General Psychopathology (16-112).

^7^ Body Mass Index (BMI): Normal: BMI<25, Overweight: 25≤BMI<30, Obese: BMI≥30.

^8^ Blood pressure (BP): Normotension = Systolic blood pressure < 140 mm Hg and Diastolic blood pressure < 90 mm Hg.

Hypertension (=Moderately high or high blood pressure) = Syst. BP ≥ 140 mm Hg and/or Diast. BP ≥ 90 mm Hg. ITT n= 94, low PUFA n=30, high PUFA n=64.

^9^ ITT n= 98, low PUFA 30, high PUFA 66.

^10^ Rbc PUFA = sum of ω-3 and ω-6 polyunsaturated fatty acids in red blood cells, µg/g rbc. For all fatty acids derived variables: patients n=97

^11^Adj. s-alpha-tocopherol = serum alpha-tocopherol /(triglycerides+cholesterol), (µmol/L)/(mmol/L), ITT n=91, low PUFA n=28, high PUFA n=61.

^12^ µmol/L, ITT n =72, low PUFA n=16, high PUFA n=54

^13^ g/L, ITT n=92, low PUFA n=27, high PUFA n=63

^14^ mmol/L, ITT n=97, low PUFA n=29, high PUFA n=66

^15^ pg/mL, ITT n=97, low PUFA n=29, high PUFA n=66

^16^ µmol/L, ITT n=95, low PUFA n=28, high PUFA n=66

**Table S3**. **Estimated changes from baseline - week 16: Symptoms, functioning, vital signs**

| **Outcome variable** | **Low PUFA patient** | | | | | **High PUFA patient** | | | | | **Treatment*Time*PUFA** | | |
| --- | --- | --- | --- | --- | --- | --- | --- | --- | --- | --- | --- | --- | --- |
|  | **β_0_** | **β_1_** | **β_2_** | **β_3_** | **β_4_** | **β_0_** | **β_1_** | **β_2_** | **β_3_** | **β_4_** | **P*T**  ***V** | **P*T**  ***E** | **P*T**  ***V*E** |
| Total PANSS | 80.8  (74.9 –  86.7) | -22.4  (-28.7 -  -16.0)  *<0.0001* | 9.2  (-2.6 -  21.0)  *0*.*12* | 12.6  (1.0 -  24.4)  *0*.*03* | -22.8  (-49.0 -  3.4)  *0*.*09* | 81.0  (76.7 - 85.2) | -22.4  (-28.7 -  -16.0)  *<0.0001* | 0.8  (-9.0 –  10.6)  *0*.*98* | -0.08  (-8.8 -  8.6)  *0*.*87* | 0.2  (-14.0 - 14.4)  *0*.*98* | *0*.*12* | *0*.*02* | *0*.*09* |
| Positive Subscale – PANSS | 18.7  (17.0 –  20.4) | -7.7  (-9.7 -  -5.6)  *<0.0001* | 5.4  (1.6 -  9.0)  *0*.*005* | 5.6  (2.0 -  9.4)  *0*.*003* | -10.0  (-18.4 -  -1.8)  *0*.*02* | 20.1  (18.9 -  21.3) | -7.7  (-9.7 -  -5.6)  *<0.0001* | -0.4  (-3.5 -  2.6)  *0*.*77* | 1.3  (-1.4 -  4.1)  *0*.*34* | 1.1  (-3.4 -  5.6)  *0*.*63* | *0*.*001* | *0*.*01* | *0*.*008* |
| P1 Delusions | 3.7  (3.2 -  4.1) | -1.6  (-2.1 -  -1.8)  *<0.0001* | 1.1  (0.7 -  2.0)  *0*.*04* | 1.2  (0.3 -  2.2)  *0*.*01* | -2.5  (-4.6 -  -0.4)  *0*.*02* | 4.0  (3.6 -  4.3) | -1.6  (-2.1 - -1.8)  *<0.0001* | -0.0  (-0.8 -  0.8)  *1*.*00* | 0.05  (-0.7 -  0.8)  *0*.*90* | 0.5  (-0.6  to 1.7)  *0*.*36* | *0*.*02* | *0*.*01* | *0*.*005* |
| P2 Conceptual disorganization | 3.2  (2.9 -  3.6) | -1.2  (-1.6 -  -0.7)  *<0.0001* | 0.7  (-0.1 -  1.6)  *0*.*09* | 0.4  (-0.5 -  1.2)  *0*.*39* | -1.0  (-2.8 -  0.8)  *0*.*30* | 3.0  (2.7 -  3.2) | -1.2  (-1.6 -  -0.7)  *<0.0001* | -0.2  (-0.9 -  0.5)  *0*.*54* | 0.1  (-0.5 -  0.7)  *0*.*78* | 0.4  (-0.6 -  1.4)  *0*.*43* | *0*.*02* | *0*.*49* | *0*.*14* |
| P3 Hallucinatory behavior | 3.3  (2.8 -  3.7) | -1.6  (-2.1 -  -1.1) *<0.0001* | 0.4  (-0.6 -  1.4)  *0*.*41* | 2.0  (0.01 -  2.0)  *0*.*048* | -1.9  (-3.3 -  1.1)  *0*.*32* | 3.5  (3.1 -  3.8) | -1.6  (-2.1 -  1.1)  *<0.0001* | 0.6  (-0.2 -  1.4)  0.12 | 0.5  (-0.2 -  1.2)  *0*.*20* | -0.8  (-2.0 -  0.3)  *0*.*17* | *0*.*62* | *0*.*27* | *0*.*82* |
| P4 Excitement | 2.2  (1.9 -  2.5) | -1.0  (-1.3 -  -0.6) *<0.0001* | 0.1  (-0.6 -  0.8)  *0*.*77* | 0.9  (0.2 -  1.6)  *0*.*02* | -0.3  (-1.8 -  1.3)  *0*.*73* | 2.3  (2.1 -  2.5) | -1.0  (-1.3 -  -0.6) *<0.0001* | -0.5  (0.6 -  0.5)  *0*.*85* | 0.6  (0.1 -  1.2)  *0*.*03* | -0.04  (-0.9 -  0.8)  *0*.*93* | *0*.*62* | *0*.*42* | *0*.*77* |
| P5 Grandiosity | 1.8  (1.4 -  2.2) | -0.7  (-1.2 -  -0.2) *0*.*006* | 0.7  (-0.1 -  1.6)  *0*.*09* | 0.8  (-0.05 -  1.6)  *0*.*06* | -1.3  (-3.2 -  0.7)  *0*.*20* | 2.4  ( 2.1 -  2.6) | -0.7  (-1.2 -  -0.2)  *0*.*006* | -0.7  (-1.4 -  0.01)  *0*.*055* | -0.2  (-0.8 -  0.4)  *0*.*56* | 0.8  (-0.1 -  2.0)  *0*.*07* | *0*.*0004* | *0*.*02* | *0*.*02* |
| P6 Suspiciousness/persecution | 3.1  (2.7 -  3.5) | -1.6  (-2.0 -  -1.1)  *<0.0001* | 1.5  (0.7 -  2.4)  *<0.0001* | 1.6  (0.7 -  2.4)  *0*.*0005* | -3.3  (-5.2 -  -1.4)  *0*.*0004* | 3.5  (3.2 -  3.7) | -1.6  (-2.0 -  -1.1)  *<0.0001* | 0.2  (-0.5 -  0.9)  *0*.*63* | 0.4  (-0.2 -  1.1)  *0*.*18* | -0.4  (-1.4 -  0.6)  *0*.*46* | *0*.*001* | *0*.*006* | *0*.*003* |
| P7 Hostility | 1.4  (1.1 -  3.3) | -0.3  (-0.6 –  0.1)  *0*.*16* | 0.9  (0.2 -  1.5)  *0*.*007* | 0.2  (-0.5 -  0.8)  *0*.*62* | -0.6  (-2.1 -  0.8)  *0*.*38* | 1.6  (1.4 -  1.8) | -0.3  (-0.6 -  0.1)  *0*.*16* | -0.1  (-0.6 -  0.4)  *0*.*61* | -0.02  (-0.5 -  0.5)  *0*.*95* | 0.5  (-0.5 -  0.5)  *0*.*48* | *0*.*001* | *0*.*57* | *0*.*21* |
| Negative Subscale – PANSS | 22.6  (20.4 –  24.9) | -3.6  (-5.8 -  -1.5)  *<0.0001* | -1.8  (-5.8 -  2.1)  *0*.*35* | 0.7  (-3.2 -  2.7)  *0*.*71* | -2.6  (-11.5 -  6.2)  *0*.*55* | 20.6  (19.0 - 22.2) | -3.6  (-5.8 -  -1.5)  *<0.0001* | -0.4  (-3.7 -  2.8)  *0*.*79* | -1.9  (-4.8 -  1.0)  *0*.*20* | 0.1  (-4.6 -  4.9)  *0*.*96* | *0*.*42* | *0*.*16* | *0*.*53* |
|  | | | | | | | | | | | | | |
|  | | | | | | | | | | | | | |
| **Outcome variable (ctd.)** | **Low PUFA patient** | | | | | **High PUFA patient** | | | | | **Treatment*Time*PUFA** | **Outcome variable** | **Low PUFA patient** |
|  | **β_0_** | **β_1_** | **β_2_** | **β_3_** | **β_4_** | **β_0_** | **β_1_** | **β_2_** | **β_3_** | **β_4_** | **P*T**  ***V** | **P*T**  ***E** | **P*T**  ***V*E** |
| General Psychopathology  Subscale – PANSS | 39.5  (36.6 -  42.4) | -10.8  (-14.1 -  -7.5)  *<0.0001* | 5.2  (-0.9 -  5.7)  *0*.*095* | 5.7  (-0.4 -  10.8)  *0*.*07* | -10.4  (-24.1 -  3.2)  *0*.*13* | 40.2  (38.1 - 42.3) | -10.8  (-14.1 -  -7.5)  *<0.0001* | 1.5  (-3.6 -  6.5)  *0*.*56* | 0.04  (-4.5 -  5.5)  *0*.*99* | -0.5  (-8.0 -  6.9)  *0*.*90* | *0*.*19* | *0*.*052* | *0*.*15* |
| G9 Unusual thought content | 3.2  (2.8 -  3.6) | -1.4  (-1.8 -  -0.9)  *<0.0001* | 1.2  (0.3 -  2.0)  *0*.*006* | 0.8  (0.001 -  1.6)  *0*.*050* | -1.6  (-3.4 -  0.1)  *0*.*07* | 3.3  (3.0 -  3.6) | -1.4  (-1.8 -  -0.9)  *<0.0001* | 0.4  (-0.3 -  1.0)  *0*.*27* | 0.2  (-0.4 -  0.8)  *0*.*43* | 0.2  (-0.8 -  1.1)  *0*.*74* | *0*.*04* | *0*.*14* | *0*.*048* |
| G15 Preoccupation | 2.1  (1.8 -  2.5) | -1.0  (-1.4 -  -0.6)  *<0.0001* | 0.8  (0.01 -  1.6)  *0*.*03* | 0.5  (-0.2 -  1.3)  *0*.*15* | 0.8  (-2.5 -  0.8)  *0*.*31* | 2.3  (2.1 -  2.6) | -1.0  (-1.4 -  -0.6)  *<0.0001* | 0.05  (-0.6 -  0.7)  *0*.*87* | 0.2  (-0.4 -  0.7)  *0*.*48* | 0.1  (-0.8 -  1.0)  *0*.*84* | *0*.*02* | *0*.*33* | *0*.*27* |
| GAF-S | 38.1  (34.8 -  41.5) | 10.5  (6.4 - 14.6)  *<0.0001* | -1.3  (8.8 -  6.3)  *0*.*74* | -1.6  (-8.9 -  5.9)  *0*.*67* | 6.2  (-10.5 -  22.9)  *0*.*46* | 35.5  (33.1 - 37.9) | 10.5  (6.4 -  14.6)  *<0.0001* | 2.8  (-3.4 –  8.9)  *0*.*38* | 2.4  (-3.1 -  8.0)  *0*.*39* | -2.4  (-10.4 -  6.7)  *0*.*61* | *0*.*25* | *0*.*26* | *0*.*31* |
| GAF-F | 38.4  (35.4 -  41.3) | 9.6  (6.2 - 12.9)  *<0.0001* | -1.8  (-8.0 -  4.3)  *0*.*56* | 1.0  (-5.1 -  7.2)  *0*.*74* | 3.3  (-10.4 -  17.0)  *0*.*64* | 36.1  (34.0 - 38.2) | 9.6  (6.2 -  12.9)  *<0.0001* | 1.0  (-4.1 -  5.6)  *0*.*71* | 3.2  (-1.3 -  7.8)  *0*.*16* | -1.9  (-9.3 -  5.6)  *0*.*62* | *0*.*33* | *0*.*46* | *0*.*46* |
| Severity of adverse events – UKU | 2.4  (1.5 -  3.3) | -0.6  (-1.6 -  0.6)  *0*.*32* | 0.1  (-1.8 -  2.0)  *0*.*89* | -1.0  (-3.0 -  0.8)  *0*.*27* | 0.04  (-4.2 -  4.2)  *0*.*98* | 2.2  (1.5 -  2.8) | -0.6  (-1.6 -  0.6)  *0*.*32* | 0.4  (-1.2 -  2.0)  *0*.*67* | -0.2  (-1.6 -  0.6)  *0*.*67* | 0.1  (-2.2 -  2.4)  *0*.*95* | *0*.*81* | *0*.*41* | *0*.*99* |
| Number of adverse events – UKU | 1.8  (1.2 -  2.4) | -0.4  (-1.0 -  0.4)  *0*.*38* | 0.02  (-1.2 -  1.2)  *0*.*98* | -0.8  (-2.2 -  0.4)  *0*.*20* | 0.2  (-2.8 -  3.0)  *0*.*92* | 1.6  (1.2 -  2.1) | -0.4  (-1.0 -  0.4)  *0*.*38* | 0.2  (-1.0 -  1.2)  *0*.*79* | -0.2  (-1.2 -  0.8)  *0*.*59* | 0.2  (-1.4 -  1.8)  *0*.*78* | *0*.*82* | *0*.*34* | *0*.*95* |
| Antipsychotic drugs – *DDD* | 1.4  (1.1 -  1.6) | -0.1  (-0.4 -  1.4)  *0*.*41* | 0.4  (-0.1 -  0.8)  *0*.*10* | 0.1  (-0.4 -  0.6)  *0*.*80* | -0.4  (-1.4 -  0.6)  *0*.*39* | 1.7  (1.5 -  1.9) | -0.1  (-0.4 -  1.4)  *0*.*41* | -0.2  (-0.6 -  0.2)  *0*.*45* | 0.1  (-0.2 -  0.4)  *0*.*66* | 0.02  (-0.4 -  0.8)  *0*.*58* | *0*.*01* | *0*.*94* | *0*.*24* |
| Blood pressure, diastolic, *mm Hg* | 75.6  (72.7 -  78.4) | 2.8  (-1.0 -  6.7)  *0*.*15* | 4.8  (-2.4 -  11.9)  *0*.*19* | 1.4  (-5.6 -  8.5)  *0*.*69* | -12.8  (-28.9 -  3.2)  *0*.*12* | 77.2  (75.0 - 79.3) | 2.8  (-1.0 -  6.7)  *0*.*15* | -3.8  (-9.5 -  2.0)  *0*.*20* | -1.6¨  (-6.9 -  3.6)  *0*.*54* | 0.2  (-8.4 -  8.8)  *0*.*97* | *0*.*01* | *0*.*37* | *0*.*11* |
| Blood pressure, systolic | 119.7  (115.8 - 123.6) | -0.7  (-6.0 -  4.7)  *0*.*80* | 9.2  (-0.6 -  19.0)  *0*.*07* | 8.1  (-1.6 -  17.9)  *0*.*10* | -15.9  (-38.0 -  6.1)  *0*.*16* | 122.9  (120.0 - 125.8) | -0.7  (-6.0 -  4.7)  *0*.*80* | -4.4  (-12.2 –  3.5)  *0*.*28* | 1.0  (-6.2 -  8.3)  *0*.*78* | 1.9  (10.0 - 13.7)  *0*.*75* | *0*.*003* | *0*.*14* | *0*.*12* |
|  | | | | | | | | | | | | | |
|  |  | | | | |  | | | | |  |  |  |
| **Outcome variable (ctd.)** | **Low PUFA patient** | | | | | **High PUFA patient** | | | | | **Treatment*Time*PUFA** | **Outcome variable** | **Low PUFA patient** |
|  | **β_0_** | **β_1_** | **β_2_** | **β_3_** | **β_4_** | **β_0_** | **β_1_** | **β_2_** | **β_3_** | **β_4_** | **P*T**  ***V** | **P*T**  ***E** | **P*T**  ***V*E** |
| Cardiac rate, beats per minute | 78.6  (74.7 -  82.5) | -0.5  (-5.5 -  4.5)  *0*.*84* | 1.4  (-7.8 -  10.6)  *0*.*76* | 2.6  (-6.6 -  11.7)  *0*.*58* | -9.6  (-30.4 -  11.2)  *0*.*36* | 77.8  (75.0 - 80.6) | -0.5  (-5.5 -  4.5)  *0*.*84* | 4.2  (-3.2 -  11.7)  *0*.*26* | -1.1  (-7.8 -  5.6)  *0*.*75* | 0.2  (-10.7 - 11.1)  *0*.*97* | *0*.*51* | *0*.*41* | *0*.*35* |
| Body Mass Index, *kg/m^2^* | 25.6  (23.9 -  27.3) | 0.7  (-0.1 -  1.4)  *0*.*09* | 0.3  (-1.1 -  1.7)  *0*.*67* | 0.3  (-1.1 -  1.7)  *0*.*64* | 0.8  (-2.4 -  4.1)  *0*.*61* | 25.4  (24.2 - 26.6) | 0.7  (-0.1 -  1.4)  *0*.*09* | 0.9  (-0.3 -  2.0)  *0*.*13* | 0.7  (-0.3 -  1.8)  *0*.*16* | -0.8  (-2.5 -  0.9)  0.33 | *0*.*36* | *0*.*53* | *0*.*30* |

**Footnote Table S3**

The linear mixed models (Model 2) have been centered on the means of red blood cells PUFA in the low (=102 µg/g RBC; **Model 3A**) and high (=441 µg/g RBC; **Model 3B**) PUFA groups, respectively. Effect coefficients: **β_0_** Intercept (estimated baseline value)**, β_1_** Time, **β_2_** Time*Vitamins, **β_3_** Time*EPA**, β_4_** Time*Vitamins*EPA. **Time**: 0 weeks=0; 16 weeks=1. **EPA**: EPA-placebo=0; EPA-active=1; **Vitamins**: Vitamins-placebo=0; Vitamins-active=1. For each effect, the estimate, the 95 % confidence interval and the p-value (except **β_0_**) are reported. In the **PUFA*Time*Treatment** columns, the p-values of interaction effects with PUFA are displayed (**P*T*V** PUFA*Time*Vitamins, **P*T*E** PUFA*Time*EPA, **P*T*V*E** PUFA*Time*Vitamins*EPA). PUFA is treated as a continuous (*not* low versus high) variable in these models. P-values are in red if <0.05, in dark red if 0.05<p-value<0.10. PUFA = Polyunsaturated fatty acids; GAF= Global Assessment of Functioning – Symptom (S) and Function (F) Scales; PANSS = Positive and Negative Syndrome Scale; UKU = UKU Side Effect Rating Scale; DDD = Defined Daily Doses. PANSS items (symptoms):

**Table S4**. **Estimated changes from baseline - week 16: Biochemical and haematological variables**

| **Outcome variable** | **Low PUFA patient** | | | | | **High PUFA patient** | | | | | **PUFA*Time*Treatment** | | |
| --- | --- | --- | --- | --- | --- | --- | --- | --- | --- | --- | --- | --- | --- |
|  | **β_0_** | **β_1_** | **β_2_** | **β_3_** | **β_4_** | **β_0_** | **β_1_** | **β_2_** | **β_3_** | **β_4_** | **P*T**  ***V** | **P*T**  ***E** | **P*T**  ***V*E** |
| Haemoglobin^1^ | 14.4  (14.0 -  14.9) | -0.2  (-0.5 -  0.1)  *0*.*21* | -0.05  (-0.6 -  0.5)  *0*.*87* | 0.1  (-0.5 -  0.6)  *0*.*82* | 0.7  (-0.5 -  2.0)  *0*.*25* | 14.4  (14.1 - 14.7) | -0.2  (-0.5 -  0.1)  *0*.*21* | 0.1  (-0.3 -  0.6)  *0*.*53* | 0.3  (-0.1 -  0.7)  *0*.*20* | -0.03  (-0.7 -  0.6)  *0*.*93* | *0*.*44* | *0*.*42* | *0*.*93* |
| Mean corpuscular volume (MCV) ^2^ | 89.3  (87.8 -  90.7) | 0.04  (-0.9 -  1.0)  *0*.*94* | -1.3  (-3.1 –  0.4)  *0*.*14* | -0.8  (-2.5 -  0.8)  *0*.*32* | -1.7  (-5.7 -  2.2)  *0*.*39* | 88.8  (87.8 - 89.8) | 0.04  (-0.9 -  1.0)  *0*.*94* | -1.1  (-.6 -  0.4)  *0*.*15* | -0.9  (-2.1 -  0.4)  *0*.*18* | 1.8  (-0.4 -  4.0)  *0*.*11* | *0*.*79* | *0*.*95* | *0*.*08* |
| Mean corpuscular haemoglobin concentration (MCHC)^3^ | 34.7  (34.3 -  35.3) | -0.1  (-0.6 -  0.3)  *0*.*56* | 0.8  (-0.5 -  1.6)  *0*.*07* | 0.2  (-0.6 -  1.0)  *0*.*62* | 1.2  (-0.7 -  3.0)  *0*.*22* | 33.8  (33.6 - 34.1) | -0.1  (-0.6 -  0.3)  *0*.*56* | 0.7  (0.005 -  1.4)  *0*.*048* | 0.4  (-0.3 -  1.0)  *0*.*26* | -0.1  (-1.1 -  1.0)  *0*.*87* | *0*.*85* | *0*.*66* | *0*.*19* |
| Leukocyte count^4^ | 7.5  (6.9 -  8.2) | -0.1  (-1.0 -  0.7)  *0*.*76* | -0.1  (-1.5 –  1.4)  *0*.*94* | -0.4  (-1.9 -  1.0)  *0*.*59* | -0.1  (-3.6 -  3.3)  *0*.*93* | 7.2  (6.9 -  7.7) | -0.1  (-1.0 -  0.7)  *0*.*76* | -0.6  (-1.9 -  0.7)  *0*.*38* | -0.7  (-1.8 -  0.4)  *0*.*20* | 0.9  (-1.0 -  2.7)  *0*.*35* | *0*.*47* | *0*.*64* | *0*.*55* |
| Platelet count^5^ | 260.0  (239.5 - 280.6) | -14.7  (-37.3 -  8.0)  *0*.*20* | 32.1  (-8.5 –  72.6)  *0*.*12* | 30.9  (-9.3 -  71.1)  *0*.*13* | -55.1  (-149.0 - 38.8)  *0*.*25* | 275.4 (260.9 - 289.9) | -14.7  (-37.3 -  8.0)  *0*.*20* | 14.9  (-19.9 - 49.7)  *0*.*40* | 3.4  (-27.4 - 34.2)  *0*.*83* | -22.9  (-73.9 - 28.1)  *0*.*38* | *0*.*37* | *0*.*15* | *0*.*50* |
| C-Reactive Protein (CRP)^6^ | 5.1  (3.5 -  6.6) | 0.3  (-1.6 -  2.1)  *0*.*78* | 1.6  (-1.8 –  4.9)  *0*.*37* | -2.0  (-5.1 -  1.1)  *0*.*20* | -5.3  (-12.7 -  2.1)  *0*.*16* | 4.2  (3.1 -  5.4) | 0.3  (-1.6 -  2.1)  *0*.*78* | -0.6  (-3.3 -  2.1)  *0*.*67* | -1.4  (-3.8 -  1.0)  *0*.*24* | -0.05  (-4.0 -  3.9)  *0*.*98* | *0*.*17* | *0*.*68* | *0*.*16* |
| Sodium^7^ | 140.6  (139.9 - 141.3) | 0.4  (-0.6 -  1.4)  *0*.*45* | 0.4  (-2.2 -  1.4)  *0*.*67* | -0.4  (-2.1 -  1.4)  *0*.*68* | -2.3  (-6.4 -  1.8)  *0*.*27* | 140.4 (139.9 - 140.9) | 0.4  (-0.6 -  1.4)  *0*.*45* | -0.3  (-1.8 -  1.3)  *0*.*75* | -0.5  (-1.8 -  0.9)  *0*.*47* | 0.3  (-1.9 -  2.6)  *0*.*79* | *0*.*87* | *0*.*88* | *0*.*21* |
| Potassium^8^ | 4.22  (4.13 -  4.31) | -0.01  (-0.15 -  0.12)  *0*.*86* | -0.21  (-0.44 -  0.02)  *0*.*07* | 0.03  (-0.19 -  0.26)  *0*.*78* | 0.14  (-0.39 -  0.67)  *0*.*60* | 4.18  (4.11 - 4.24) | -0.01  (-0.15 -  0.12)  *0*.*86* | 0.08  (-0.12 - 0.29)  *0*.*43* | -0.004  (-0.18 - 0.17)  *0*.*97* | -0.09  (-0.38 - 0.20)  *0*.*54* | *0*.*01* | *0*.*75* | *0*.*39* |
| Calcium (albumin adjusted)^9^ | 2.32  (2.28 -  2.36) | -0.03  (-0.09 -  0.03)  *0*.*27* | 0.02  (-0.08 -  0.11)  *0*.*76* | 0.05  (-0.05 -  0.14)  *0*.*34* | -0.04  (-0.26 -  0.19)  *0*.*75* | 2.35  (2.32 - 2.38) | -0.03  (-0.09 -  0.03)  *0*.*27* | 0  (-0.09 - 0.09)  *0*.*99* | 0.003  (-0.07 - 0.08)  *0*.*93* | -0.03  (-0.15 - 0.10)  *0*.*69* | *0*.*74* | *0*.*35* | *0*.*92* |
| Creatinine^10^ | 79.9  (76.1 -  83.8) | 3.6  (0.1 -  7.1)  *0*.*046* | -6.8  (-13.2 -  -0.4)  *0*.*04* | -4.2  (-10.6 -  2.3)  *0*.*20* | 0.7  (-12.0 -  13.6)  *0*.*91* | 79.5  (76.7 - 82.2) | 3.6  (0.1 -  7.1)  *0*.*046* | -4.0  (-9.5 -  1.5)  *0*.*16* | -3.3  (-8.2 -  1.5)  *0*.*18* | -1.0  (-7.0 -  4.9)  *0*.*73* | *0*.*34* | *0*.*77* | *0*.*46* |
| Uric acid^11^ | 313.8  (278.9 - 348.7) | -2.9  (-38.2 -  32.4)  *0*.*87* | -11.2  (-81.9 -  59.6)  *0*.*76* | 68.1  (0.7 -  135.4)  *0*.*048* | -72.2  (-208.4 - 64.0)  *0*.*30* | 312.3 (291.1 - 333.4) | -2.9  (-38.2 -  32.4)  *0*.*87* | -4.8  (-54.5 - 44.9)  *0*.*85* | 18.0  (-27.2 - 63.3)  *0*.*43* | -28.4  (-98.5 - 41.6)  *0*.*42* | *0*.*83* | *0*.*10* | *0*.*51* |
|  |  | | | | |  | | | | |  | | |
| **Outcome variable (ctd.)** | **Low PUFA patient** | | | | | **High PUFA patient** | | | | | **PUFA*Time*Treatment** | | |
|  | **β_0_** | **β_1_** | **β_2_** | **β_3_** | **β_4_** | **β_0_** | **β_1_** | **β_2_** | **β_3_** | **β_4_** | **P*T**  ***V** | **P*T**  ***E** | **P*T**  ***V*E** |
| Albumin^12^ | 41.3  (40.0 -  42.6) | 2.4  (1.1 -  3.8)  *0*.*001* | -0.9  (-3.4 -  1.5)  *0*.*46* | 0.7  (-1.8 -  3.2)  *0*.*57* | 1.1  (-4.6 -  6.8)  *0*.*70* | 42.6  (41.7 - 43.5) | 2.4  (1.1 -  3.8)  *0*.*001* | -1.5  (-3.6 -  0.7)  *0*.*18* | -1.2  (-3.1 -  0.7)  *0*.*20* | 1.9  (-1.2 -  5.0)  *0*.*24* | *0*.*65* | *0*.*10* | *0*.*80* |
| Ferritin^13^ | 97.3  (68.7 -  125.8) | -14.9  (-43.4 -  13.6)  *0*.*30* | -9.3  (-60.2 -  41.6)  *0*.*72* | 15.3  (-33.6 -  64.2)  *0*.*54* | 31.7  (-81.6 - 145.0)  *0*.*58* | 88.7  (68.4 - 109.0) | -14.9  (-43.4 -  13.6)  *0*.*30* | -5.4  (-48.0 - 37.1)  *0*.*80* | -3.2  (-41.0 - 34.7)  *0*.*87* | -8.8  (-70.4 - 52.8)  *0*.*78* | *0*.*87* | *0*.*40* | *0*.*47* |
| Alanine aminotransferase (ALAT)^14^ | 36.7  (25.8 -  47.5) | -8.7  (-22.6 -  5.3)  *0*.*22* | 5.1  (-19.2 -  29.4)  *0*.*68* | 20.2  (-4.7 -  45.2)  *0*.*11* | -35.0  (-91.3 -  21.3)  *0*.*22* | 39.8  (32.3 - 47.5) | -8.7  (-22.6 -  5.3)  *0*.*22* | 0.6  (-20.4 - 21.6)  *0*.*98* | 7.3  (-11.5 - 26.1)  *0*.*44* | -6.0  (-36.7 - 24.6)  *0*.*70* | *0*.*66* | *0*.*12* | *0*.*23* |
| Gamma-glutamyl transferase  (γ-GT)^15^ | 34.1  (26.2 -  42.1) | -4.4  (-13.2 -  4.5)  *0*.*33* | 10.6  (-5.5 -  26.7)  *0*.*20* | 1.8  (-14.0 -  17.6)  *0*.*82* | -12.8  (-49.4 -  23.8)  *0*.*49* | 32.8  (27.3 - 38.4) | -4.4  (-13.2 -  4.5)  *0*.*33* | 11.5  (-2.0 -  25.1)  *0*.*09* | 4.0  (-7.9 - 15.9)  *0*.*50* | -5.4  (-25.1 - 14.4)  *0*.*59* | *0*.*90* | *0*.*77* | *0*.*69* |
| Cholesterol^16^ | 5.40  (5.05 -  5.75) | 0.11  (-0.20 -  0.42)  *0*.*48* | 0.11  (-0.48 -  0.70)  *0*.*70* | 0.03  (-0.52 -  0.58)  *0*.*91* | 0.62  (-0.70 -  1.93)  0.35 | 5.04  (4.79 - 5.29) | 0.11  (-0.20 -  0.42)  *0*.*48* | 0.53  (0.02 - 1.04)  *0*.*04* | 0.26  (-0.19 - 0.69)  *0*.*26* | -1.20  (-1.93 - -0.48)  *0*.*001* | *0*.*17* | *0*.*40* | *0*.*008* |
| Triglycerides^17^ | 2.00  (1.61 -  2.40) | -0.14  (-0.60 -  0.32)  *0*.*54* | 0.08  (-0.79 -  0.94)  *0*.*86* | -0.19  (-1.00 -  0.61)  *0*.*64* | -0.10  (-2.02 -  1.83)  *0*.*92* | 1.69  (1.40 - 1.97) | -0.14  (-0.60 -  0.32)  *0*.*54* | 0.67  (-0.06 - 1.40)  *0*.*07* | 0.36  (-0.28 - 1.00)  *0*.*27* | -0.87  (-1.93 - 0.16)  *0*.*10* | *0*.*17* | *0*.*16* | *0*.*42* |
| Glucose^18^ | 5.30  (5.13 -  5.47) | 0.13  (-0.09 -  0.35)  *0*.*24* | 0.29  (-0.11 -  0.70)  *0*.*15* | -0.32  (-0.69 -  0.06)  *0*.*10* | -0.25  (-1.15 -  0.65)  *0*.*59* | 5.07  (4.95 - 5.38) | 0.13  (-0.09 -  0.35)  *0*.*24* | 0.02  (-0.32 - 0.36)  *0*.*91* | 0.08  (-0.22 - 0.38)  *0*.*58* | -0.28  (-0.77 - 0.21)  *0*.*26* | *0*.*17* | *0*.*03* | *0*.*95* |
| Free thyroxin^19^ | 15.0  (14.0 -  16.0) | -0.5  (-1.7 -  0.8)  *0*.*44* | -1.3  (-3.6 -  0.9)  *0*.*25* | 0.6  (-1.4 -  2.7)  *0*.*54* | 2.5  (-2.4 -  7.5)  *0*.*31* | 15.0  (14.3 - 15.7) | -0.5  (-1.7 -  0.8)  *0*.*44* | -1.7  (-3.5 -  0.2)  *0*.*08* | -0.2  (-1.8 -  1.4)  *0*.*81* | 2.3  (-0.4 -  5.0)  *0*.*10* | *0*.*73* | *0*.*39* | *0*.*92* |
| Thyroidea stimulating hormone (TSH) ^20^ | 2.10  (1.56 -  2.63) | -0.38  (-1.14 -  0.37)  *0*.*32* | 1.01  (-0.32 -  2.35)  *0*.*13* | 0.28  (-0.98 -  1.54)  *0*.*67* | -1.12  (-4.07 -  1.82)  *0*.*45* | 2.22  (1.86 - 2.58) | -0.38  (-1.14 -  0.37)  *0*.*32* | 0.91  (-0.20 - 2.02)  *0*.*11* | 0.43  (-0.55 - 1.41)  *0*.*39* | -0.69  (-2.30 - 0.91)  *0*.*40* | *0*.*87* | *0*.*80* | *0*.*77* |
| Total antioxidant capacity^21^ | 1.36  (1.30 -  1.41) | -0.03  (-0.10 -  0.05)  *0*.*47* | 0.02  (-0.11 -  0.16)  *0*.*74* | 0.03  (-0.10 -  0.16)  *0*.*70* | 0.03  (-0.27 -  0.34)  *0*.*83* | 1.33  (1.29 - 1.37) | -0.03  (-0.10 -  0.05)  *0*.*47* | 0.04  (-0.07 - 0.16)  *0*.*47* | 0.003  (-0.10 - 0.11)  *0*.*95* | -0.01  (-0.18 - 0.16)  *0*.*91* | *0*.*77* | *0*.*73* | *0*.*78* |
|  | | | | | | | | | | | | | |
|  | | | | | | | | | | | | | |
| **Outcome variable (ctd.)** | **Low PUFA patient** | | | | | **High PUFA patient** | | | | | **PUFA*Time*Treatment** | | |
|  | **β_0_** | **β_1_** | **β_2_** | **β_3_** | **β_4_** | **β_0_** | **β_1_** | **β_2_** | **β_3_** | **β_4_** | **P*T**  ***V** | **P*T**  ***E** | **P*T**  ***V*E** |
| F2-isoprostane^22^ | 37.8  (31.1 -  44.6) | -0.35  (-10.4 -  9.7)  *0*.*95* | 2.7  (-16.0 -  21.5)  *0*.*77* | 3.5  (-13.6 -  20.5)  *0*.*69* | 26.9  (-13.6 -  67.3)  *0*.*19* | 36.3  (31.4 - 41.2) | -0.35  (-10.4 -  9.7)  *0*.*95* | -7.7  (-22.7 - 7.3)  *0*.*31* | 3.6  (-9.6 - 16.9)  *0*.*59* | -4.9  (-26.8 - 17.0)  *0*.*66* | *0*.*24* | *0*.*98* | *0*.*12* |
| Malondialdehyde (TBARS)^23^ | 0.45  (0.40 -  0.50) | 0.02  (-0.04 -  0.08)  *0*.*48* | -0.05  (-0.16 -  0.07)  *0*.*41* | -0.03  (-0.13 -  0.08)  *0*.*63* | 0.14  (-0.16 -  0.44)  *0*.*37* | 0.47  (0.43 - 0.50) | 0.02  (-0.04 -  0.08)  *0*.*48* | -0.04  (-0.14 - 0.05)  *0*.*40* | 0.02  (-0.06 - 0.10)  *0*.*65* | 0.003  (-0.14 - 0.14)  *0*.*97* | *0*.*93* | *0*.*37* | *0*.*36* |
| S-alpha-tocopherol  (lipid adjusted)^24^ | 4.2  (3.3 -  5.0) | -0.2  (-1.3 -  0.9)  *0*.*67* | 4.9  (2.9 -  6.9)  *<0.0001* | 0.9  (-1.0 -  2.8)  *0*.*35* | -1.6  (-6.1 -  2.8)  *0*.*47* | 6.1  (5.5 -  6.7) | -0.2  (-1.3 -  0.9)  *0*.*67* | 6.3  (4.6 -  8.0) *<0.0001* | 0.5  (-1.0 -  2.0)  *0*.*47* | -0.1  (-2.6 -  2.4)  *0*.*93* | *0*.*16* | *0*.*71* | *0*.*50* |
| RBC PUFA^25^ | 110.6  (80.7 -  140.6) | 39.2  (-7.9 -  86.3)  *0*.*10* | 66.3  (-14.8 - 147.5)  *0*.*11* | 229.9  (147.9 - 312.0) *<0.0001* | -302.1  (-485.2 -  -119.0)  *0*.*001* | 437.2 (415.4 - 459.1) | 39.2  (-7.9 -  86.3)  *0*.*10* | -52.0  (-120.5 - 16.4)  *0*.*14* | 19.8  (-39.7 - 79.4)  *0*.*51* | -10.2  (-110.7 - 90.3)  *0*.*84* | *0*.*003* | *<0.0001* | *0*.*002* |
| ω-6/ ω-3 ratio | 6.2  (5.5 -  6.8) | -1.1  (-2.1 -  0.1)  *0*.*03* | -1.2  (-2.9 -  0.5)  *0*.*16* | -2.7  (-4.3 -  1.0)  *0*.*002* | 3.7  (-0.01 -  7.4)  *0*.*06* | 4.0  (3.5 -  4.4) | -1.1  (-2.1 -  0.1)  *0*.*03* | 0.6  (-0.9 -  2.0)  *0*.*44* | 0.4  (-1.7 -  0.8)  *0*.*49* | -0.6  (-2.6 -  1.5)  *0*.*60* | *0*.*03* | *0*.*007* | *0*.*03* |
| Eicosapentaenoic acid (EPA) ^25^ | 2.1  (-1.8 -  6.1) | 5.1  (-1.1 -  11.3)  *0*.*11* | 3.7  (-7.0 -  14.4)  *0*.*50* | 24.2  (13.4 -  35.0) *<0.0001* | -28.6  (-52.7 -  4.4)  *0*.*02* | 11.7  (8.8 -  14.6) | 5.1  (-1.1 -  11.3)  *0*.*11* | -0.4  (-9.4 -  8.7)  *0*.*94* | 21.0  (13.2 -  28.8) *<0.0001* | 5.3  (-8.0 -  18.5)  *0*.*43* | *0*.*43* | *0*.*55* | *0*.*006* |
| Docosahexaenoic acid (DHA) ^25^ | 9.4  (3.1 -  15.6) | 6.3  (-2.5 -  15.1)  *0*.*16* | 15.4  (-0.2 -  30.9)  *0*.*053* | 30.6  (14.8 -  46.3) *<0.0001* | -48.5  (-84.1 -  13.0)  *0*.*008* | 55.7  (51.2 - 60.2) | 6.3  (-2.5 -  15.1)  *0*.*16* | -6.1  (-19.4 -  7.1)  *0*.*36* | -1.1  (-12.6 - 10.4)  *0*.*86* | -5.4  (-24.9 - 14.2)  *0*.*59* | *0*.*004* | *<0.0001* | *0*.*02* |
| Arachidonic acid (ARA) ^25^ | 24.9  (14.3 -  35.5) | 13.3  (-3.0 -  29.5)  *0*.*11* | 19.4  (-8.9 -  47.7)  *0*.*18* | 65.0  (36.4 -  93.6) *<0.0001* | -87.1  (-151.1 -  -23.1)  *0*.*008* | 136.8 (129.1 - 144.5) | 13.3  (-3.0 -  29.5)  *0*.*11* | -18.7  (-42.6 - 5.2)  *0*.*12* | -9.0  (-29.7 - 11.8)  *0*.*40* | -13.6  (-48.7 - 21.5)  *0*.*45* | *0*.*005* | *<0.0001* | *0*.*03* |

**Footnote Table S4.** See Table S3 for an explanation of the linear mixed models.

*Units of variables:* ^1^ g/100 mL blood, ^2^ fL, ^3^ g/100 mL, ^4^ 10^9^/L, ^5^ 10^9^/L, ^6^ mg/L, ^7^ mmol/L, ^8^ mmol/L, ^9^ mmol/L, ^10^ µmol/L, ^11^ µmol/L, ^12^ g/L, ^13^ µg/L, ^14^ U/L, ^15^ U/L, ^16^ mmol/L, ^17^ mmol/L, ^18^ mmol/L, ^19^ nmol/L, ^20^ mU/L, ^21^ mmol/L, ^22^ pg/mL, ^23^ µmol/L, ^24^ (µmol/L)/(mmol/L), ^25^ µg/g rbc ^2^ 3
